# Implementation strategies for evidence-based management of type 2 diabetes at primary healthcare facilities in low- and middle-income countries and related barriers and facilitators: a scoping review

**DOI:** 10.1186/s43058-026-00998-9

**Published:** 2026-06-16

**Authors:** Asima Khan, Izhan Ali Khan, Khalid Rehman, Gerardo Zavala Gomez, Imogen Featherstone, Kamran Siddiqi, Saima Afaq

**Affiliations:** 1 Department of Health Sciences, University of York, York, UK; 2 Primary Care Diabetes Association, Karachi, Pakistan; 3 Institute of Social Sciences and Public Health, Khyber Medical University, Peshawar, Pakistan; 4 Hull York School, University of York, York, UK

**Keywords:** Implementation strategies, Expert recommendations for implementing change (ERIC), Consolidated framework for implementation research (CFIR), Diabetes mellitus, Low-Middle income countries (LMICs), Primary healthcare

## Abstract

**Background:**

Type 2 Diabetes prevalence is rapidly increasing in low- and middle-income countries (LMICs), where constrained health budgets and inequitable resource distribution limit access to quality care. Primary healthcare is central to addressing these challenges; however, the implementation of evidence-based diabetes care remains inconsistent. This scoping review mapped implementation strategies for integrating diabetes care into primary healthcare settings in LMICs and identified associated barriers and facilitators influencing implementation.

**Methods:**

This review focuses on type 2 diabetes, given its predominance and relevance to primary healthcare delivery in LMICs, and followed the Joanna Briggs Institute methodology for scoping reviews and was reported in accordance with PRISMA-ScR guidelines. The search strategy was peer-reviewed using the PRESS checklist. Eight electronic databases were searched for studies published between January 1996 and November 2024. Eligible studies were conducted in LMICs, as defined by the World Bank, and described the implementation, adaptation, or evaluation of evidence-based Type 2 diabetes care in primary healthcare settings. Two reviewers independently screened titles, abstracts, and full texts. Implementation strategies were mapped to the Expert Recommendations for Implementing Change (ERIC) taxonomy, while reported barriers and facilitators were coded using the Consolidated Framework for Implementation Research (CFIR).

**Results:**

Ninety-two articles describing 85 studies across 27 LMICs were included. Implementation strategies most frequently clustered within *Engage Consumers*, *Change Infrastructure*, and *Use Evaluative and Iterative Strategies*, whereas *Adapt and Tailor to Context* and *Utilise Financial Strategies* were less often explicitly reported. Forty-three studies reported clinical outcomes only, 11 reported implementation outcomes only, and 31 reported both. Studies more frequently reported improvements in clinical and/or implementation outcomes that combined multiple strategies, particularly provider training and decision support alongside patient or family engagement and pragmatic system redesign. Co-occurrence analyses indicated that *Train and Educate Stakeholders* was frequently paired with *Engage Consumers*, supported by supervision, feedback mechanisms, and infrastructure strengthening. CFIR mapping suggested that workflow integration, leadership engagement, regular supervision, and reliable medicine and diagnostic supplies were commonly associated with improved adoption and fidelity, while connectivity challenges, stock-outs, and high workload disrupted implementation and limited scale-up. Reporting of adoption, fidelity, and acceptability remained uneven, and financial strategies were poorly described despite their relevance to sustainability.

**Conclusion:**

In LMIC primary healthcare settings, diabetes care implementation most commonly emphasises provider training, infrastructure strengthening, and interactive support strategies, while explicit attention to contextual adaptation and financial mechanisms is less frequently reported. Implementation outcomes were reported to be influenced by leadership engagement, digital tools, and community involvement, alongside persistent constraints related to workforce capacity, supply chains, and feedback systems. Future implementation efforts should more explicitly address contextual fit, system integration, and sustainability when designing and scaling diabetes care interventions in primary healthcare.

**Supplementary information:**

The online version contains supplementary material available at 10.1186/s43058-026-00998-9.

Contributions to the literature

- This scoping review provides a comprehensive synthesis of implementation strategies used to deliver evidence-based type 2 diabetes care in primary healthcare settings across low- and middle-income countries
- By applying ERIC and CFIR frameworks, it offers a structured understanding of how implementation strategies and contextual determinants interact in resource-constrained settings
- The review identifies key gaps in the reporting of contextual adaptation, financial strategies, and implementation outcomes, highlighting priorities for designing and scaling sustainable diabetes care interventions in LMIC primary healthcare

## Introduction

### Background

Low- and middle-income countries (LMICs) face persistent inequities in healthcare, with public sector resources often insufficient to address the growing burden of non-communicable diseases such as diabetes [1]. Limited healthcare workforce capacity, infrastructure constraints, and high reliance on out-of-pocket expenditure, accounting for approximately 55.4% of total health spending, further exacerbate these challenges [2, 3]. Addressing such disparities requires a strong focus on primary healthcare (PHC). The World Health Organisation has repeatedly emphasised PHC as the cornerstone of equitable, accessible, and cost-effective healthcare, as articulated in the Alma-Ata Declaration and reaffirmed in the Astana Declaration [4, 5].

Diabetes is a chronic and progressive condition with rapidly increasing prevalence worldwide, disproportionately affecting LMICs [6]. Approximately 80% of people living with diabetes reside in LMICs, and an estimated 26.9% of adults with diabetes remain undiagnosed [7, 8]. Without timely diagnosis and effective management, diabetes may lead to serious and life-threatening complications such as cardiovascular disease, stroke, kidney failure, blindness, and lower-limb amputation, resulting in a decreased quality of life and increased healthcare costs [9]. Evidence-based guidelines clearly outline effective components of diabetes care, including multidisciplinary management, patient empowerment, and adherence to clinical protocols [2, 10]. Implementing these essential components requires a set of strategies that can redefine the functions of healthcare providers and promote the recipient’s self-management [11]. However, access to such evidence-based care remains limited in many LMIC settings.

PHC is increasingly recognised as the most appropriate platform for the prevention, screening, and long-term management of diabetes and other chronic conditions, particularly in resource-constrained settings [12]. While numerous interventions have suggested effectiveness under controlled conditions, translating these interventions into routine PHC practice remains challenging in LMICs. These challenges are less about the absence of clinical evidence and more about how evidence-based diabetes care is implemented, integrated, and sustained within real-world health systems. In this review, evidence-based diabetes care is conceptualised as a composite of interventions addressing glycaemic control, blood pressure and lipid management, screening and management of complications, and patient self-management support, consistent with World Health Organisation recommendations for diabetes care in primary healthcare [13]. This operational definition is important as diabetes care comprises multiple components with varying levels of complexity, resource requirements, and expected benefit.

Implementation strategies refer to the systematic methods used to support the adoption, integration, and sustainability of evidence-based interventions within healthcare settings. The Expert Recommendations for Implementing Change (ERIC) taxonomy provides a structured framework for categorising such strategies across nine clusters, including training and education, consumer engagement, system redesign, and evaluative approaches [14]. These strategies may target patients, healthcare providers, or organisational and system-level processes [15]. Understanding how these strategies have been applied in LMIC PHC settings, and the barriers and facilitators influencing their implementation, is critical for strengthening diabetes care delivery.

Despite the growing application of diabetes interventions in LMIC primary care, implementation strategies remain inconsistently described and poorly synthesised across the literature. LMICs were the focus of this review due to the disproportionate burden of diabetes and the unique health system constraints affecting implementation in these settings. To date, there has been no comprehensive review systematically mapping the strategies used to implement evidence-based diabetes care in LMIC PHC settings and examining the contextual factors influencing their uptake.

Therefore, this scoping review aims to systematically identify, describe, and classify implementation strategies used for delivering diabetes management in primary healthcare settings in LMICs using the ERIC framework, and to summarise reported barriers and facilitators to implementation to inform future implementation planning.

### Aims of the study

This scoping review aimed to:

1. Systematically identify studies describing the implementation of evidence-based diabetes care within primary healthcare settings in low- and middle-income countries (LMICs).
2. Describe and classify the implementation strategies used to support diabetes care in LMIC primary healthcare, using the Expert Recommendations for Implementing Change (ERIC) framework.
3. Summarise reported barriers and facilitators influencing the implementation of diabetes care in primary healthcare settings, drawing on the Consolidated Framework for Implementation Research (CFIR) to support structured interpretation.
4. Provide an overview of reported implementation and clinical outcomes associated with these strategies.

## Methods

### Study design and framework

This scoping review was conducted in accordance with the Joanna Briggs Institute (JBI) methodology for scoping reviews [16] and adheres to the PRISMA-ScR (Preferred Reporting Items for Systematic Reviews and Meta-Analyses extension for Scoping Reviews) reporting guidelines. The review approach was underpinned by the methodological framework proposed by Arksey and O’Malley, which comprises six stages: (1) identifying the research question; (2) identifying relevant studies; (3) defining study selection criteria; (4) charting the data; (5) collating, summarising, and reporting the results; and (6) optionally consulting with stakeholders or peers to enhance interpretation and validate findings [17]. All core stages of this framework were followed in conducting the review. While formal stakeholder consultation was not undertaken as a discrete study phase, iterative discussions with supervisors and peers informed interpretation of findings and refinement of the synthesis, consistent with the consultative intent of the framework. The protocol for this scoping review is preregistered at the Open ScienceFramework DOI **10.17605/OSF.IO/WCT5J**

### Identifying the relevant studies

#### Search strategy

The search strategy for Medline (Ovid) was developed in collaboration with an experienced librarian at the University of York, following the Peer Review of Electronic Search Strategies (PRESS) guidelines [18]. This strategy was subsequently adapted for use with other databases. A three-stage process was followed, as described in the JBI guidelines. (Annexure 1). The search was initiated in November 2024 in Medline (Ovid), Cochrane Central Register of Controlled Trials (CENTRAL), PubMed, Scopus, Web of Science, and Embase (Ovid). To capture grey literature, additional searches were conducted on ProQuest and Google Scholar, including policy documents, reports, dissertations, and conference proceedings, using verbatim and advanced search options. The first five pages of results were reviewed after sorting by relevance. The publications identified through the search were imported into Covidence software (available at http://www.covidence.org) for screening and management.

### Eligibility criteria

We used the Population, Concept, and Context Framework to identify appropriate literature for this scoping review [19]. which guided the identification and selection of relevant studies.

Population: Studies were eligible if they involved individuals with Type 2 Diabetes Mellitus (T2DM) of any age. Studies focusing exclusively on Type 1 diabetes, gestational diabetes, or pre-diabetes were excluded.

Concept: Eligible studies explicitly described the use of one or more implementation strategies to support the delivery of evidence-based diabetes care. The included studies reported how strategies were applied in practice and/or described barriers and facilitators influencing their uptake, delivery, or sustainability. Studies reported only clinical outcomes without reference to implementation strategies or processes were excluded.

Context: Studies were included if conducted in primary healthcare (PHC) settings, such as community health centres, clinics, health posts, or basic health units, operating in either the public or private sector, undertaken in low- and middle-income countries (LMICs). Country income classification was based on the World Bank classification corresponding to the period in which each study was published. Given that the majority of included studies were published within the last decade, the potential impact of temporal changes in country classification is likely minimal [20, 21].

Studies conducted solely at the community level without linkage to formal healthcare facilities were excluded. All empirical study designs were eligible, including qualitative, quantitative, and mixed-methods studies. Economic evaluations, intervention development studies without implementation, and clinical practice guideline development papers were excluded. Publications in any language were considered. The search period spanned January 1996 to November 2024, reflecting the period during which evidence-based diabetes management became established in the early 1990s, followed by the emergence of implementation science to address research-to-practice gaps [22, 23].

### Study selection

**Fig. 1:**
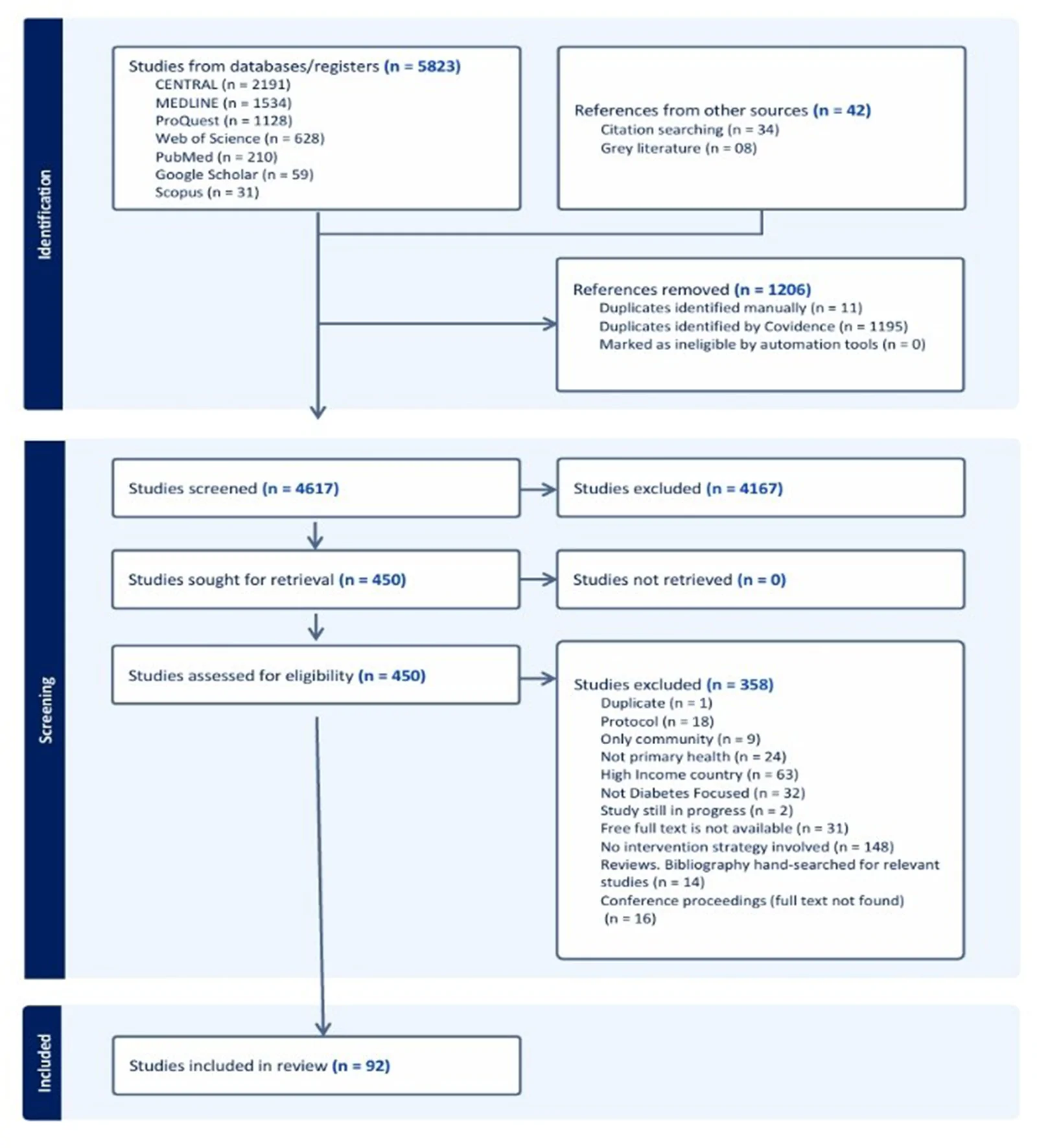
PRISMA flow diagram of study selection

Two independent authors (AK, SI, or IAK) screened the titles and abstracts of the studies identified in our search. After careful selection, we retrieved the full texts of all eligible studies for inclusion. Full-text screening for eligibility was conducted independently by two reviewers (AK as the primary reviewer, and either SI or IAK as the second reviewer). Any disagreements regarding study selection were resolved through discussion, with consultation from a senior researcher when necessary to reach a final decision. Studies published in languages other than English were eligible for inclusion. Where potentially relevant non-English studies were identified, initial screening was conducted using translated titles and abstracts. Four studies met the inclusion criteria for full-text screening: two Spanish, one Chinese and one Portuguese. Translation was initially conducted using machine translation tools. For the one included non-English study, translation accuracy was subsequently verified by a native Spanish speaker to ensure fidelity of interpretation. Any study excluded was justified, and the reasons were recorded. If a study was only available as an abstract or under a paywall, we consulted the authors where possible and excluded the study if a free full text was unavailable. We hand-searched the bibliography at the full-text screening stage for relevant studies. (Fig. 1)

### Data extraction

A pre-specified and piloted data extraction log was used. It was created in Covidence to ensure systematic charting of key variables. A 50% double-extraction approach was used, which is consistent with methodological guidance for scoping reviews, and their results were compared to assess consistency. Minor discrepancies were resolved through discussion with a senior researcher. As agreement was found to be high, the remaining data extraction was completed by AK (Annexure 2).

### Data analysis

Extracted data were analysed using a combination of descriptive, framework-based, and narrative synthesis approaches. Descriptive statistics were used to summarise the characteristics of included studies, including geographical location, study design, and type of primary healthcare setting.

Implementation strategies were systematically mapped to the Expert Recommendations for Implementing Change (ERIC) taxonomy [14]. Each intervention was assigned a dominant ERIC cluster, defined as the strategy that represented the primary aim of the intervention; moreover, additional strategies were recorded to reflect the multi-component nature of many programmes. This approach allowed examination of both the distribution of dominant strategies and common strategy combinations across studies.

All reported barriers and facilitators were coded using the Consolidated Framework for Implementation Research (CFIR) [24]. These were organised across CFIR’s five domains, intervention characteristics, outer setting, inner setting, characteristics of individuals, and implementation processes, to support a structured interpretation of how contextual and organisational factors influenced implementation.

Reported implementation outcomes (including adoption, fidelity, feasibility, acceptability, and sustainability) and clinical outcomes (e.g. HbA1c, fasting blood glucose, blood pressure) were extracted in line with Proctor’s implementation outcomes framework [25] Outcomes were not pooled or formally compared across studies.

Outcomes were interpreted descriptively to identify high-level signals of success or challenge. Interventions were considered to show positive outcome signals when studies reported improvements in clinical or implementation outcomes, mixed signals when findings were inconsistent or context-dependent, and limited or unclear signals when outcomes were not reported or showed minimal change (Table 1). These interpretations were based on the authors’ reported findings and were not intended to imply causal effects.

**Table 1:** Studies were grouped by World Bank income categories to facilitate interpretation

S.No	Intervention Name	Citation & Country	Type of Primary Healthcare	Main & Secondary ERIC strategies	Delivered by	mode of delivery	duration of the intervention	*Outcomes Implementation/Clinical/Qualitative	**Reported Effectiveness
	**Low-income countries**								
1	Training and implementation of the Ethiopian Primary Healthcare Clinical Guidelines (EPHCG) for NCD and mental Healthcare.	[26] Ethiopia	Health Facilities/Multi-Centre	**Train and Educate Stakeholders;** Support clinicians; Provide interactive assistance; Use evaluative and iterative strategies	Ministry of Health (MoH), regional health bureaus.	On-site training, mentoring.	not specified	Implementation	NA
2	Nutrition-promotion programme to improve dietary adherence and glycaemic control among people with type 2 diabetes in Ethiopia.	[27] Ethiopia	Four public hospitals/Multi-Centre	**Engage Consumers;** Train and educate stakeholders; Develop stakeholder interrelationships	Two health promoters + one facilitator	Educational sessions (group & individual) + written materials	6 months	Clinical and implementation	Positive
3	Family medicine registrar–rural health centre partnership model to strengthen primary care management of diabetes and hypertension in Malawi	[28] Malawi	Rural health centre/Single Centre	**Provide Interactive Assistance;** Change infrastructure; Support clinicians; Train and educate stakeholders; Develop stakeholder interrelationships; Adapt and tailor to context	Family Medicine registrars, local staff Supported by undergraduate students	On-site teaching, mentorship, WhatsApp support, follow-up visits	Ongoing over 20 months	Implementation	Positive
4	Integrated management system for hypertension and diabetes care.	[29] Tanzania	District hospital + 10 Health Centres/Multi-Centre	**Change Infrastructure;** Support clinicians; Change policy context; Develop stakeholder interrelationships; Adapt and tailor to context	Tosamaganga DDH + 10 health centers staff	Structured pathway with registration, monthly follow-ups, and reassessment at 6 months	6-month follow-up with ongoing implementation	Clinical and Implementation	Mixed
5	Integrated Care Package (ICP) Grid – Framework for Evaluating Implementation of Integrated Diabetes and Hypertension Care	[30] Cambodia (Belgium, Slovenia)	Health Centres (HCs) and Referral Hospitals (RHs)/Multicenter	**Use Evaluative and Iterative Strategies;** Change infrastructure; Support clinicians; Train and educate stakeholders; Engage Consumers; Develop stakeholder interrelationships	Public sector facilities (HCs/RHs) and community-based peer educators (MoPoTsyo)	Facility-based care (treatment, education), community-based peer support	Ongoing implementation (snapshot assessment during 2019–2022)	Implementation	NA
	**Lower-middle-income countries**								
6	mPower Heart Project – task-sharing model with mobile decision-support system.	[31] India	Community health centres/Multi-Centre	**Supporting Clinician;** Change infrastructure; Train and educate stakeholders; Develop stakeholder interrelationships; Adapt and tailor to context	Medical officers and Nurses	mobile phone	21 months	Clinical	Positive
7	CARRS multicomponent quality-improvement strategy for sustained diabetes risk-factor control in South Asian primary care.	[32] India and Pakistan	public and private urban diabetes clinics/Multi-Centre	**Support Clinician;** Train and educate stakeholders; Use evaluative and iterative strategies	Clinicians non-physician care coordinators	in person- face to face	6.5 years	Clinical and Implementation	Mixed
8	Comprehensive Diabetes Management Programme (CDMP) group-based education and self-care support to improve glycaemic control and quality of life.	[33] India	Urban Health Training Centre/Single Centre	**Engage Consumers;** Train and educate stakeholders; Develop stakeholder interrelationships	Investigators and medical social workers.	Group-based peer support sessions. One-on-one educational counselling. Family involvement in counselling.	6 months	Clinical and Implementation	Positive
9	An integrated diabetes and hypertension case management package	[34] Bangladesh	Two primary care facilities/Multi-Centre	**Change Infrastructure;** Train and educate stakeholders; Engage Consumers; Use evaluative and iterative strategies	Trained PHC doctors + Nurses	Clinical guide + Patient record system + Training workshops	3 months	Clinical and implementation	Mixed
10	Implementation status of the non-communicable disease control program	[35] Bangladesh	Upazila Health Complexes (UzHCs) - PHC facilities/Multi-Centre	**Use Evaluative and Iterative Strategies;** Change infrastructure; Support clinicians; Change policy context;	Doctors, nurses, and paramedics at UzHCs	Facility-based screening, diagnosis, and treatment at NCD corners	Ongoing implementation	Implementation	NA
11	Team-Based Chronic Care Model to strengthen diabetes and hypertension management through task redistribution and staff capacity building in rural India	[36], [37] India	Public primary Healthcare centers (PHCs)/Multi-Centre	**Change Infrastructure;** Support clinicians; Train and educate stakeholders; Develop stakeholder interrelationships	Medical doctors, nurses, lab technicians, pharmacists, and Accredited Social Health Activists (ASHAs)	PHC-based service reorganisation ASHA involvement in patient follow-up (not consistently implemented)	9 months	Clinical and Implementation	Mixed
12	Quality improvement initiative involving a redesigned diabetic foot care pathway	[38] India	Dispensary/Single Centre	**Use Evaluative and Iterative Strategies;** Train and educate stakeholders; Engage Consumers; Change policy context	Primary care doctors, nurses, surgeons	In-person foot exams, dedicated diabetes clinics	6 months (April to Sept 2017)	Implementation-focused Qualitative	NA
13	Improving access to medicines for non-communicable diseases	[39] India	Rural Primary Health Centres (PHCs)/Multi-Centre	**Change Infrastructure;** Support clinicians; Train and educate stakeholders; Engage Consumers; Develop stakeholder interrelationships	PHC staff with project facilitation	PHC visits, monthly monitoring,	18 months	Clinical and implementation	Negative
14	Comprehensive Primary Healthcare (CPHC) Strengthening Intervention – Ayushman Bharat Model HWCs	[40] India	Subcenter-level Health and Wellness Centers (SC-HWCs)/Multi-Centre	**Change Infrastructure;** Support clinicians; Train and educate stakeholders; Engage Consumers; Develop stakeholder interrelationships; Adapt and tailor to context	Community Health Officers (CHOs), MPWs, PHC team	Facility-based service improvement + community mobilization + patient groups (Fateh groups)	6 months follow-up	Clinical and implementation	Positive
15	Electronic Medical Record (EMR) Implementation for Chronic Disease Management	[41] India	Rural community health centers/Multi-Centre	**Change Infrastructure;** Support clinicians; Train and educate stakeholders; Engage Consumers; Adapt and tailor to context	Community doctors linked to super-specialty hospital	Face-to-face consultation, EMR-based documentation	3 months (pilot implementation)	Implementation	Positive
16	Digital Decentralised Care Model (Upazila Health Complex Intervention)	[42] Bangladesh	subdistrict NCD clinic and community clinics/Multicenter	**Change Infrastructure;** Train and educate stakeholders; Engage Consumers	Physician, community paramedic & community health worker	Screening/follow-up at village clinics; treatment initiation/titration at subdistrict NCD clinics.	6 months	Clinical and implementation	Positive
17	mPower Heart – mobile-based clinical decision-support system (mDSS) for nurse-facilitated screening and management of diabetes in primary care.	[43], [44] India	5 Community Health Centres (CHCs)/Multi-Centre	**Support Clinician;** Change infrastructure; Train and educate stakeholders; Use evaluative and iterative strategies	Nurse Care Coordinators (NCCs) + Physicians.	Nurses assessed patients & entered data into mDSS. Physicians reviewed & approved treatment plans.	18 months.	Clinical and Implementation	Positive
18	Médecins Sans Frontières (MSF) model of care for diabetes and hypertension management in refugee settings in Lebanon	[45] Lebanon	MSF NCD Clinic/Single Centre	**Change Infrastructure;** Support clinicians; Train and educate stakeholders; Engage Consumers; Develop stakeholder interrelationships	General practitioners & nurses for case management. Health promoters for education and counselling. Psychologists for mental health integration.	Task-shifting approach: Nurses and doctors provided interchangeable consultations. Comprehensive same-day visits (consultation, lab tests, medication refill, and counselling).	6 months follow-up.	Clinical and implementation	Positive
19	Medication Adherence Clubs (MACs)	[46] Kenya	Primary Healthcare clinic/Single Centre	**Engage Consumers;** Support clinicians; Use evaluative and iterative strategies	Nurses facilitated the clubs, clinical officers reviewed yearly or as needed	Group sessions (25 to 35 patients), quarterly meetings	Ongoing, with quarterly reviews	Clinical and Implementation	Positive
20	Evaluation of Integrated Diabetes Care Package	[47] Pakistan	Public primary Healthcare facilities/Multi-Centre	**Use evaluative and iterative strategies**; Train and Educate Stakeholders; Engage Consumers	Medical doctors and allied Healthcare staff at primary Healthcare facilities	Routine outpatient services at public health facilities	9 months	Clinical and implementation	Mixed
21	First Line Diabetes Care (FILDCARE) Project	[48] Philippines	Local government health units (LGHUs)/Multi-Centre	**Train and Educate Stakeholders;** Support clinicians; Engage Consumers	Local government health unit staff (doctors, nurses, midwives, barangay health workers)	Routine consultations with embedded self-management education and support (SME/S)	1 yr	Clinical and Implementation	Positive
22	Clinical Practice Guideline (CPG) Adherence for Diabetes Care	[49], [50] Palestine	Primary Healthcare Centres/Multi-Centre	**Use Evaluative and Iterative Strategies**	PHC doctors		On going	Qualitative	Not Applicable
23	eHealth Netbook Application for Screening and Referral Support	[51] Lebanon	5 PHC/Multi-Centre	**Change Infrastructure;** Develop stakeholder interrelationships	Trained Community Health Workers (CHWs).	In-person screening + automated referrals.	6 months (2015).	Clinical **and** implementation	Positive
24	Mobile Health (mHealth) Messaging Intervention for Chronic Disease Self-Management Developed in Partnership with MoPoTsyo Patient Information Centre	[52] Cambodia	Community-based, NGO-supported peer education system/Multi-Centre	**Use Evaluative and Iterative Strategies;** Support clinicians; Train and educate stakeholders; Engage Consumers	The intervention was delivered via pre-recorded voice messages, initiated by the research team (University of Washington and MoPoTsyo)	Pre-recorded voice calls (not SMS), sent to mobile phones of participants.	Pilot phase delivered messages twice weekly; full intervention planned as ongoing, repetitive support	Implementation	Unclear
25	Implementation of clinical guidelines for diabetes and hypertension management	[53, 54] Mongolia	10 family health centres/Multi-Centre	**Develop Stakeholder Interrelationships;** Change infrastructure; Train and educate stakeholders; Engage Consumers; Use evaluative and iterative strategies	Primary care providers (doctors and nurses).	Training sessions, provision of resources, and monitoring.	Ongoing since 2011.	Qualitative	NA
26	REaCH – Remote Consulting for Health Training and Mobile-Supported Implementation for Chronic Disease Management	[55] Tanzania and Nigeria	Public, faith-based, and private primary care facilities/Multicenter	**Change Infrastructure;** Support clinicians; Train and educate stakeholders	Tier 1 & 2 trained health workers (nurses, clinical officers, etc.)	Phone-based consultations (feature phones/smartphones)	9-month rollout (staggered by sequence)	Clinical and implementation	Positive
	**Studies conducted across mixed-income country settings**								
27	Digital messaging to support control for type 2 diabetes (StAR2D)	[56] South Africa and Malawi	Primary Care Clinic/Multi-Centre	**Engage Consumers;** Train and educate stakeholders	Automated SMS system	4 SMS per week (12 months)		Clinical and Implementation	Mixed
28	Text Message Diabetes Self-Management Support (DSMS)	[57] DR Congo, Cambodia, Philippines	Public and NGO-supported diabetes programs/Multicenter	**Engage Consumers;** Train and educate stakeholders; Develop stakeholder interrelationships; Adapt and tailor to context	Educators, managers, supported by NGO/clinic teams	SMS (5/week in DR Congo, 6/week in Cambodia, 2/week in Philippines)	2 years	Clinical and Implementation	Mixed
	**Upper-middle-income Countries**								
29	Health-literacy and health-belief–based group education with follow-up phone counselling	[58] Turkey	Primary Care Unit/Single Centre	**Engage Consumers;** Train and educate stakeholders; Adapt and tailor to context	Nurses & trained educators.	weekly face-to-face sessions for six weeks, followed by phone counselling for six weeks	12 weeks	Clinical and Implementation	Mixed
30	Diabetic foot screening quality improvement cycle	[59] South Africa	Primary Healthcare clinics/Single Centre	**Train and Educate Stakeholders;** Support clinicians; Engage Consumers	Clinic Healthcare workers	In person- through Staff training sessions, patient education	one year	implementation	Positive
31	Community-based diabetes education programme involving patients, families, and primary Healthcare providers.	[60] Costa Rica	Five of the ten Basic Primary Healthcare Teams (EBAIS)/Multi-Centre	**Engage Consumers;** Train and educate stakeholders; Develop stakeholder interrelationships	Trained Healthcare providers at primary care clinics	Training sessions for Healthcare workers Group education sessions for patients	4 months of structured education, followed by sustainability activities	Clinical and Implementation	Positive
32	Simpler Tool – structured clinical guideline to improve diabetes care delivery and glycaemic control.	[61] Malaysia	Primary health clinic/Multi-Centre	**Support Clinician;** Train and educate stakeholders; Engage Consumers	Pharmacists trained in using the Simpler tool	Pharmacist-led patient education and medication review Structured follow-up visits	6 months	Clinical	Positive
33	mHealth-based diabetes registry and decision-support tool to improve quality of care in primary health centres.	[62] Argentina	20 public primary care centres in Corrientes, Argentina/Multi-Centre	**Engage Consumers;** Change infrastructure	Primary care teams (physicians, nurses, community health workers)	Data collection during clinical encounters, decision support prompts	1 year	Clinical and Implementation	Unclear
34	Health-coaching intervention using motivational interviewing to improve glycaemic, psychosocial, and self-care outcomes in adults with diabetes.	[63] China	41 Community Health Stations in Fengtai District, Beijing/Multi-Centre	**Provide Interactive Assistance;** Train and educate stakeholders; Engage Consumers; Change policy context	Community doctors, nurses, and psychologists trained as health coaches	Face-to-face and telephone coaching sessions	12 months	Clinical and Implementation	Mixed
35	Nurse training on educational actions for diabetes care within primary Healthcare settings.	[64] Brazil	Basic Health Unit/Single Centre	**Train and Educate Stakeholders**	Nurses in Primary Care	Face-to-face interactions, group meetings, home visits	Ongoing (part of regular Primary Care)	Qualitative	NA
36	Implementation of staged diabetes management (SDM) protocols to standardise diabetes care in Brazilian primary health settings.	[65] Brazil	Two municipalities in Bahia/Multi-Centre	**Change Infrastructure;** Train and educate stakeholders; Change policy context	Healthcare professionals (doctors, nurses, pharmacists, health technicians) trained in SDM protocols	Training sessions, regular updates, and case discussions	18 months	Clinical and implementation	Positive
37	Culturally adapted lifestyle intervention using the trans-cultural diabetes-specific nutrition algorithm (tDNA) with MI	[66] Malaysia	Primary care clinic/Single Centre	**Engage Consumers**	Dietitian and physician	Face-to-face counseling	6 months	Clinical and implementation	Positive
38	Will Diabetes Care Move Closer to Rural Patients in China?	[67] China	township health centres/Multi-Centre	**Develop Stakeholder Interrelationships;** Train and educate stakeholders	Service team (physicians, nurses, public healtphysicians, diabetes specialists)	Face-to-face lectures, home visits, and special medical services	2 years (2015–2017)	Clinical and implementation	Mixed
39	InsuOnline – a serious educational game to train primary care physicians in insulin therapy.	[68] Brazil	Primary Care/Multi-Centre	**Develop Stakeholder Interrelationships;** Support clinicians; Use evaluative and iterative strategies	Self-directed Game	Participants downloaded and installed the game from a website, with login credentials provided.	Average time to complete the game: ~4 hours.	Implementation	Positive
40	Contextually adapted self-management care-plan booklet used during diabetes and hypertension consultations in South Africa	[69] South Africa	Three community Healthcare facilities: urban CHC, rural CHC, urban PHC clinic/Multi-Centre	**Engage Consumers;** Train and educate stakeholders; Develop stakeholder interrelationships	Providers (doctors, nurses, health promoters)	Integrated into consultations	One month	Qualitative	NA
41	Pictorial diary handbook (PDHB) programme to support diabetes self-management and improve glycaemic control in primary care.	[70] Thailand	Primary Care Unit/Multi-Centre	**Engage Consumers;** Train and educate stakeholders; Develop stakeholder interrelationships	Primary Care Providers	Weekly group education + group activity training + individual checklist reviews + monthly home visits	3 months	Clinical and implementation	Positive
42	eHealth family-based education programme delivered via WeChat to improve glucose control and self-care among adults with diabetes.	[71] China	Community health service center/Single Centre	**Engage Consumers;** Train and educate stakeholders; Develop stakeholder interrelationships	Community health center staff	WeChat articles + In-person sessions	12 months	Clinical and implementation	Positive
43	An effective system of nurse-led diabetes care in rural Africa	[72] South Africa	Hlabisa Hospital and 14 peripheral Primary Healthcare clinics/Multi-Centre	**Support Clinician;** Change infrastructure; Train and educate stakeholders	Two nurses (one British Diabetes Specialist Nurse and one local nurse).	Weekly hospital clinics and monthly PHC clinics.	18 months	Clinical and Implementation	Positive
44	Peer support with diabetes self-management education to improve glycaemic control and quality of life	[73] Mexico	Community Health Centre/Single Centre	**Engage Consumers;** Train and educate stakeholders; Develop stakeholder interrelationships; Adapt and tailor to context	Trained peer leaders (PLs) + Certified diabetes educator	Peer-led group sessions + Community meetings	8 months	Clinical and implementation	Positive
45	mHealth-enabled hierarchical diabetes management programme (ROADMAP) to improve glycaemic control in primary care in China.	[74] China	864 communities across 25 provinces in China/Multi-Centre	**Use Evaluative and Iterative Strategies;** Support clinicians; Train and educate stakeholders; Engage Consumers	Primary care doctors, county hospital doctors.	Monthly clinic visits with structured diabetes care. Automated alerts for abnormal blood glucose or BP. Quarterly performance review of primary care doctors.	12 months.	Clinical and implementation	Positive
46	Telemedicine-based intervention for diabetes education, advancement, and support to enhance self-management and patient experience	[75], [76] Malaysia	Ministry of Health primary care clinics/Multi-Centre	**Engage Consumers;** Change infrastructure; Support clinicians; Train and educate stakeholders	Research team & physicians	Bluetooth data upload + clinician interface	6 months (nested in IDEAS trial)	Clinical and implementation	Negative
47	Culturally adapted, evidence-based diabetes self-management programme for Chinese adults using insulin therapy	[77] China	Public community health service centre/Single Centre	**Engage Consumers;** Train and educate stakeholders; Develop stakeholder interrelationships; Adapt and tailor to context	First author (trained nurse)	Face-to-face sessions + phone follow-up	6 sessions over 6 weeks	Clinical and implementation	Positive
48	Group-visit model for diabetes self-management support in rural communities of Shanghai.	[78] China	Community health centers/Multi-Centre	**Engage Consumers;** Support clinicians; Train and educate stakeholders; Develop stakeholder interrelationships	GP, nurse, preventive doctor, peer group leader	Group sessions + individual consults at end of session	12 monthly group sessions (1.5 hrs + 1 hr one-on-one)	Clinical and Implementation	Positive
49	Family doctor system implemented to improve continuity and coordination of diabetes care in urban China.	[79] China	Community Health Centres (CHCs)/Multi-Centre	**Provide Interactive Assistance;** Change infrastructure; Support clinicians; Engage Consumers; Change policy context	Family doctor teams (GP, nurse, public health physician)	Primary care-based outpatient and health management services	Continuous registration over 3 years (2015â€“2017)	Implementation	Positive
50	Guideline-based clinical decision tree for diabetes management using real-world primary care data in China.	[80] China	Primary care and registry-based outpatient settings/Multi-Centre	**Provide Interactive Assistance;** Change infrastructure; Support clinicians; Train and educate stakeholders; Change policy context	Primary care physicians using decision tree outputs	Outpatient-based, registry-integrated prescription decisions	3-month outcomes measured	Clinical and implementation	Positive
51	Web-based Clinical Decision Support System (CDSS) for DM/HTN	[81] Brazil	Family Health Strategy (FHS) primary health centres/Multi-Centre	**Support Clinician;** Change infrastructure; Train and educate stakeholders; Adapt and tailor to context	Primary care teams (doctors, nurses, CHWs)	Digital patient care planning, alerts, teleconsultation support	6 months formal pilot; data collected over ~2 years (2016â€“2018)	Implementation and qualitative	Unclear
52	Group-based educational nursing intervention designed to promote self-care among older adults with diabetes mellitus.	[82] Brazil	Primary Healthcare Units/Multi-Centre	**Engage Consumers;** Train and educate stakeholders	Researcher (nursing professional)	Group sessions (average 5 participants/session)	60-minute session + 15-day follow-up call	Clinical and implementation	Positive
53	Group diabetes education program delivered by health promoters through four monthly sessions at public community health centers in South Africa	[83] South Africa	Community Health Centers/Multi-Centre	**Train and Educate Stakeholders;** Engage Consumers	Health promoters (lay workers trained over 6 days)	Group sessions (60 mins/session)	4 sessions over 4 months	Clinical and implementation	Mixed
54	Group Empowerment and Training (GREAT) program	[84], [85] South Africa	Primary Care Facilities/Multi-Centre	**Train and educate stakeholders**; Engage Consumers: Use Evaluative and Iterative Strategies	Trained facilitators (nurses, health promoters, dieticians).	4 group sessions (diabetes basics, lifestyle, medication, complications).	Sessions spaced over weeks/months.	Qualitative	Not Applicable
55	Redesigned diabetes care model using an interprofessional team approach.	[86] Ecuador	Rural primary care practices/Multi-Centre	**Change Infrastructure;** Support clinicians; Train and educate stakeholders; Engage Consumers; Use evaluative and iterative strategies	Nurse case manager, pharmacist, dietitian	In-person, team-based visits (30â€“60 mins)	36 months (4 visits/year)	Clinical	Positive
56	Comprehensive diabetes program integrating digital health, capacity building, and guideline-based care.	[87] Argentina	Public primary care centres/Multi-Centre	**Train and Educate Stakeholders;** Change infrastructure; Support clinicians; Engage Consumers; Adapt and tailor to context	Physicians, nurses, CHWs, peers	In-person + digital tools	Up to 24 months follow-up	Clinical and implementation	Mixed
57	Health in Every Hut” community-based diabetes and hypertension follow-up program using trained outreach workers	[88] South Africa	Community-based outreach in 10 rural clinic catchments/Multi-Centre	**Change Infrastructure;** Support clinicians; Train and educate stakeholders; Engage Consumers; Develop stakeholder interrelationships; Adapt and tailor to context	Trained Community Health Outreach Workers (CHOWs)	Weekly in-home follow-up visits	Median 6 visits per patient over 2+ years	Clinical	Positive
58	ACE Program – Analytical and Clinical Excellence Point-of-Care Testing (POCT) initiative for diabetes management.	[89] South Africa	Urban primary care diabetes clinic/Single Centre	**Change Infrastructure;** Support clinicians; Train and educate stakeholders; Engage Consumers; Adapt and tailor to context	Trained clinic staff (local health workers, nurses)	On-site POCT, immediate clinical decision-making	Avg. 15 months follow-up (repeat POCTs, up to 5 per patient)	Clinical + Implementation	Positive
59	SMS Text Messaging for Diabetes Care Strengthening Program	[90] Argentina	Public primary care clinics (20 PCCs in low-resource settings)/Multi-Centre	**Use Evaluative and Iterative Strategies;** Train and educate stakeholders; Engage Consumers; Develop stakeholder interrelationships; Adapt and tailor to context	Automated system (messages developed with primary care teams)	One-way SMS weekly via a web-based platform	12 months (message period before follow-up)	Implementation and Qualitative	NA
60	Tshwane Insulin Project (TIP) – Nurse-led, app-enabled insulin management program	[91] South Africa	Public primary care clinics and home-based outreach (Tshwane District)/Multi-Centre	**Provide Interactive Assistance;** Change infrastructure; Support clinicians; Train and educate stakeholders; Engage Consumers; Develop stakeholder interrelationships; Adapt and tailor to context	Nurses, CHWs, medical officers	Clinic-based nurse initiation; home follow-up and insulin titration via app by CHWs	Weekly CHW home visits; monthly clinic follow-up (ongoing)	Implementation and Qualitative	Unclear
61	Nutrition education and individual dietary counselling program on metabolic control	[92] Iran	Community health centers in Andimeshk, Iran/Multi-Centre	**Train and Educate Stakeholders;** Engage Consumers; Adapt and tailor to context	Two nutritionists	Group sessions (3 - 90 min) + individual counselling (2 - 45 min)	4 months follow-up	Clinical	Positive
62	Family Doctor Concept (FDC) – Primary Care Restructuring Model	[93] Malaysia	Public primary health clinics/Multi-Centre	**Change Infrastructure;** Support clinicians; Train and educate stakeholders; Engage Consumers	Primary Healthcare Teams (assigned by zones)	Team-based care, geographic zone assignment	Not applicable (cross-sectional assessment)	Clinical and implementation	Positive
63	Daily SMS-Based Diabetes Education Program	[94] South Africa	Outpatient departments of PHCs/Multi-Centre	**Train and Educate Stakeholders;** Engage Consumers	Automated system (Zoomconnect platform)	Unidirectional SMS (1/day for 6 months)	6 months		
64	DIAPREM – Diabetes Primary Care, Registry, Education, and Management Program	[95] Argentina	Public primary Healthcare units/Multi-Centre	**Use Evaluative and Iterative Strategies;** Change infrastructure; Support clinicians; Train and educate stakeholders; Engage Consumers; Develop stakeholder interrelationships	Trained physicians and nurses	Quarterly clinic visits + reminder calls + structured data collection	12 months	Clinical and implementation	Positive
65	EMPOWER-PAR – Multistrategic Diabetes Care Model Based on the Chronic Care Framework	[96] Malaysia	Public Primary Care Clinics/Multi-Centre	**Change Infrastructure;** Support clinicians; Train and educate stakeholders; Use evaluative and iterative strategies	Multidisciplinary Chronic Disease Management (CDM) team (doctors, nurses, pharmacists, dietitians).	Team-based care, patient education, and self-management support.	1 year	Clinical and Implementation	Positive
66	Brief Motivational Interviewing (Telephone + In-Person) for Diabetes Self-Management	[97] Thailand	Health Promoting Hospital/Single Centre	**Engage Consumers;** Support clinicians; Train and educate stakeholders; Adapt and tailor to context	Trained nurse (MI-certified, study author)	Week 4 & 8 in-person; Weeks 5 & 7 via phone	8 weeks total (Weeks 0â€“4: usual care, Weeks 4â€“8: MI)	Clinical and Implementation	Mixed
67	Community Health Center Models for Chronic Disease Management (Public, Gatekeeper, and Hospital-Managed CHCs)	[98] China	Community Health Centres (CHCs)/Multi-Centre	**Use Evaluative and Iterative Strategies;** Change infrastructure; Change policy context; Adapt and tailor to context	Structured care, coordinated system			Clinical and Implementation	Positive
68	DIABEMPIC – Multicomponent Integrated Care Program for Diabetes Empowerment and Improved Glycaemic Control	[99] Mexico	Public primary outpatient Healthcare centres + a specialised diabetes clinic/Multi-Centre	**Change Infrastructure;** Support clinicians; Train and educate stakeholders; Engage Consumers; Use evaluative and iterative strategies	Multidisciplinary team: endocrinologists, nurses, dietitians, psychologists, etc.	Group and individual education, shared medical appointments	5 months (26 hours total)	Clinical and implementation	Positive
69	Primary Diabetes Care Using Vichai’s Seven-Colour Balls Model	[100] Thailand	Sub-district health promotion hospitals (SHPH)/Multi-Centre	**Use Evaluative and Iterative Strategies;** Change infrastructure; Train and educate stakeholders; Engage Consumers; Develop stakeholder interrelationships	Nurses, public health officers, VHVs (village health volunteers)	In-person clinic visits, home visits for high-risk patients	Ongoing (routine care)	clinical and implementation	Mixed
70	Motivational Interviewing (MI) in Individual Nursing Consultations for Diabetes and Hypertension Management	[101] Brazil	Family health units under Public Health System/Multi-Centre	**Provide Interactive Assistance;** Support clinicians; Train and educate stakeholders; Engage Consumers	Trained nurses (20 hrs MI)	Face-to-face consultations	2 sessions, 30â€“50 min each	Clinical and Implementation	Positive
71	Structured Clinical Record (SR) Incorporating National Guidelines for Diabetes and Hypertension Management	[102] South Africa	Community Health Centres (CHCs)/Multi-Centre	**Change Infrastructure;** Support clinicians; Train and educate stakeholders; Use evaluative and iterative strategies	Clinic staff (nurses and doctors) after outreach education by local experts	Paper record inserted into patient folders; used during routine visits	12 months	Clinical and implementation	Negative
72	IMB-Based Behavioural Program to Slow Chronic Kidney Disease Progression in Patients with Diabetes and Hypertension	[103] Thailand	Subdistrict Health Promoting Hospitals (rural community-based PHC)/Multicenter	**Engage Consumers;** Support clinicians; Train and educate stakeholders; Develop stakeholder interrelationships; Adapt and tailor to context	Public health nurses and researchers	Group sessions + mobile app + phone calls	14 weeks	Clinical and Implementation	Positive
73	Printed Patient Decision Aid (PDA) for Shared Decision-Making on Insulin Initiation in Primary Care	[104] Malaysia	Public community clinics, private clinics, and university hospital/Multicenter	**Engage Consumers;** Support clinicians; Train and educate stakeholders; Develop stakeholder interrelationships; Adapt and tailor to context	seven consultations by five clinicians: one general practitioner (private clinic) and four medical officers (public clinics and hospital)	Distributed before consultation; referred to during consultation	Varies per consultation; data collected over 5 months	Qualitative	NA
74	Casalud Model – Innovative Healthcare Model for Prevention and Control of Non-Communicable Diseases	[105] Mexico	Primary Health Clinics (PHCs)/Multicenter	**Use Evaluative and Iterative Strategies;** Change infrastructure; Train and educate stakeholders; Change policy context; Adapt and tailor to context	PHC staff (doctors, nurses, social workers), supported by FundaciÃ^3^ n Carlos Slim	Facility- and community-based NCD screening and management with tech support	Ongoing; assessed over a 2-year implementation period	Implementation and Clinical	Positive
75	Motivational Interviewing (MI)-Based Community Health Agent–Led Diabetes Self-Management Support Program	[106] Brazil	Basic Health Unit/Single Centre	**Engage Consumers;** Train and educate stakeholders; Develop stakeholder interrelationships; Adapt and tailor to context	Community health agents (*n* = 19)	Monthly home visits	6 months	Clinical and implementation	Positive
76	Implementation Intention–Based Behavioural Adherence Intervention	[107] Brazil	Two public primary Healthcare units/Multicenter	**Train and Educate Stakeholders;** Engage Consumers; Adapt and tailor to context	Primary researcher, guided participants through action/coping plans	4 face-to-face sessions + 2 phone follow-ups	15 weeks	Clinical and Implementation	Positive
77	Mobile Screening and Individualised Treatment Feedback Intervention	[108] South Africa	Government-run PHC clinics (provincial/local) and8community health centres/Multicenter	**Support Clinician;** Change infrastructure; Train and educate stakeholders; Engage Consumers; Use evaluative and iterative strategies; Adapt and tailor to context	Mobile unit + expert review panel	On-site mobile unit screening and feedback	12 months	Clinical and implementation	Mixed
78	Community-Based Diabetes Program with Continuity Enhancement	[109] China	Community Health Centres (CHCs), urban Shanghai	**Change Infrastructure;** Support clinicians; Train and educate stakeholders; Engage Consumers; Use evaluative and iterative strategies; Develop stakeholder interrelationships	Trained family doctors (CHC)	Group education + routine consultations	9 months	Clinical and Implementation	Positive
79	Community Health Worker (CHW) Accompaniment Model	[110] Mexico	Government-supported rural health centers/Multicenter	**Provide Interactive Assistance;** Change infrastructure; Train and educate stakeholders; Engage Consumers; Develop stakeholder interrelationships	Community Health Workers (CHWs) local women trained by CES	Regular home visits (weekly to monthly), clinic accompaniment	12 months post-rollout in each community	Clinical and implementation	Positive
80	Physician–Pharmacist Collaborative Clinic Model	[111], [112] China	Primary Healthcare centers/Multicenter	**Develop Stakeholder Interrelationships;** Support clinicians; Train and educate stakeholders; Engage Consumers	Physicians (usual care) + pharmacists (medication guidance, education, WeChat group management).		12 months with follow-ups at 3, 6, 9, and 12 months.	Implementation	NA
81	China’s National Essential Public Health Service Package (NEPHSP)	[113] China	CHCs and CH stations (urban) and township HCs and village clinics (rural)/Multicenter	**Use Evaluative and Iterative Strategies;** Change infrastructure; Support clinicians; Train and educate stakeholders	physicians			Qualitative and Implementation	NA
82	Family Doctor Contract Service Model	[114] China	community health centers and hospitals/Multicenter	**Develop Stakeholder Interrelationships;** Engage Consumers; Change policy context	Family doctors (teams with nurses/public health practitioners).	In-person (CHCs), phone calls, home visits.	Ongoing (2015–2017).	Clinical and implementation	Positive
83	SMARTDiabetes Digital Health Intervention	[115] China		**Develop Stakeholder Interrelationships;** Utilise financial strategies; Change infrastructure; Support clinicians; Train and educate stakeholders; Change policy context	PHC provider			Clinical and implementation	Positive
84	Nudge Strategy–Based Dietary Education Program	[116] China	Primary care practices/Multicenter	**Develop Stakeholder Interrelationships;** Engage Consumers	Nurses and endocrinologists	Face-to-face, group sessions	4 weeks, weekly sessions	Clinical	Positive
85	Peer Leader Support Program (PLSP)	[117] China	Community Health Centers (CHCs) in Chengdu/Multicenter	**Engage Consumers,** Train and educate stakeholders; Develop stakeholder interrelationships	Peer leaders + CHSC staff	Group meetings, informal activities Group meetings, informal activities	6 months	Clinical and Implementation	Positive

Findings from the ERIC and CFIR analyses were integrated through narrative synthesis. For each ERIC cluster, results were synthesised to describe the types of studies included, the outcomes most commonly reported, and key barriers and facilitators influencing implementation across CFIR domains.

Tables and heat maps were used to illustrate the number and proportion of studies by ERIC cluster and region. This combined analytical approach provided both a structural overview of how implementation strategies were applied and a context-sensitive interpretation of why certain approaches appeared more feasible, acceptable, or sustainable for strengthening diabetes care in LMIC primary healthcare settings

## Results

A total of 5,823 studies were identified, of which 4,620 remained after duplicate removal and were included in the title and abstract screening initiated on 27 November 2024. Out of these, 450 studies were eligible for full-text screening. Ninety-two articles representing 85 distinct programmes were included (Table 1), spanning 27 LMICs with the largest contributions from China (*n* = 17), South Africa (*n* = 10), and India (*n* = 8); five were multinational (Annexure 3). The Western Pacific, African, and South-East Asian regions contributed over two-thirds of the evidence. By World Bank classification, upper-middle-income settings accounted for 58%, lower-middle-income 30%, and low-income 12%. Most included studies were conducted after 2015, reducing the likelihood of misclassification due to temporal changes in country income status. The study setting was explicitly reported in 30 of 85 studies (35%). Among these, 15 were conducted in rural settings, 10 in urban settings, and 5 in mixed settings, indicating that a substantial proportion of studies did not specify context.

Designs included RCTs (*n* = 23, 27.1%), mixed-methods (*n* = 10, 11.8%), qualitative (*n* = 9, 10.6%), quasi-experimental (*n* = 8, 9.4%), and observational studies. Seventy studies (82.4%) were multi-centre, and 15 (17.6%) were single-centre. Primary care delivery models varied across government facilities, outreach, and digital platforms (family physicians, nurses, pharmacists, and community health workers), reflecting heterogeneous systems and resources. Barriers were reported in 78 of 92 studies (84.8%), and facilitators in 75 of 92 (81.5%), while 72 studies (78.3%) reported both. CFIR mapping highlighted context and organisational readiness as key influencers to implementation. A full study listing, including settings, designs, scope, and dominant ERIC clusters, is presented in Table 1.

### Implementation strategies mapped to the ERIC framework

**Fig. 2:**
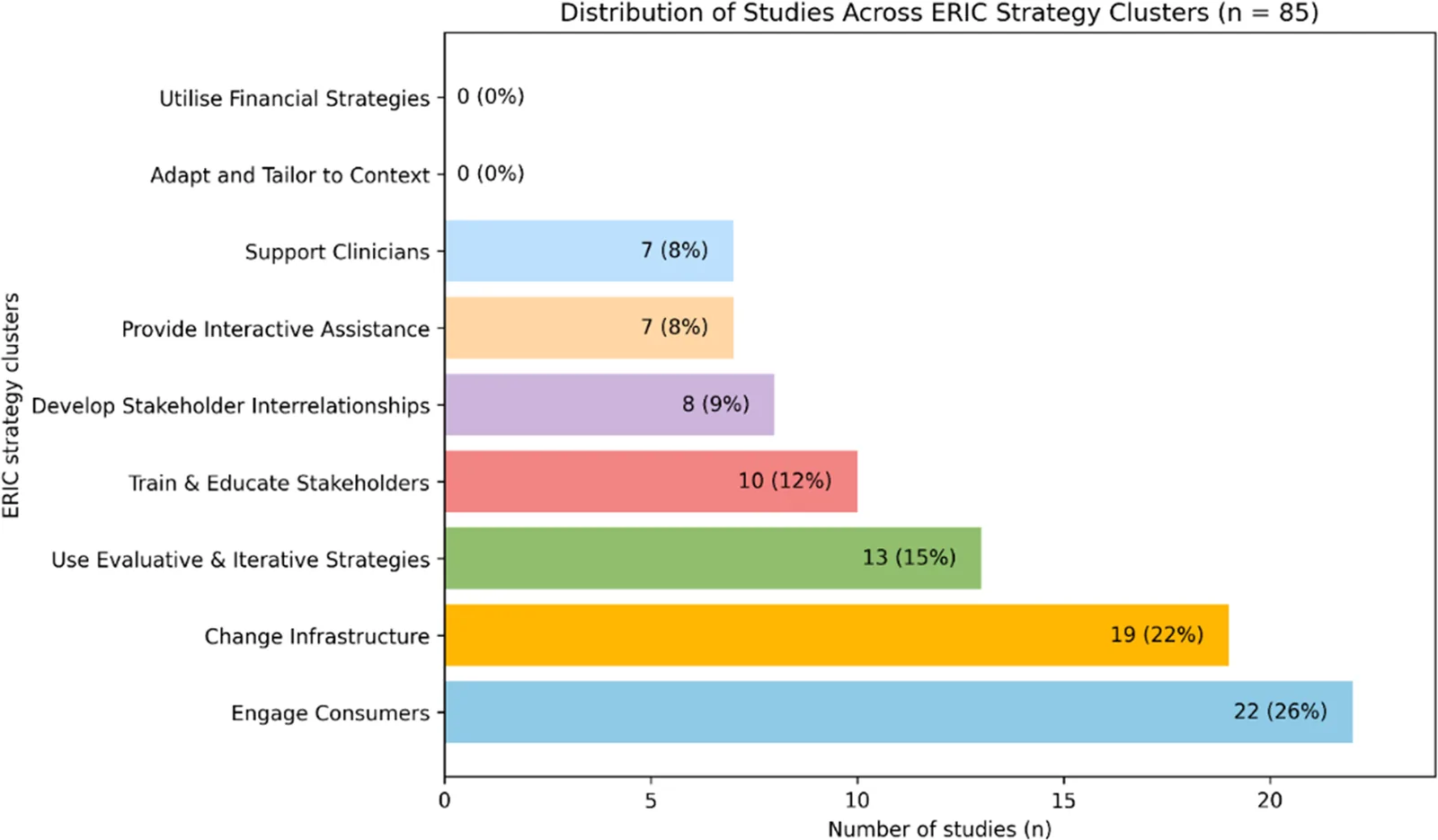
Distribution of included studies by ERIC cluster counts as per the dominant strategy used

When considering the dominant implementation strategy (i.e. the strategy that constituted the primary aim of the intervention), most studies focused on ‘Engage Consumers’, ‘Change Infrastructure’, or ‘Use Evaluative and Iterative Strategies’ (Fig. 2). ‘Adapt and Tailor to Context’ and ‘Utilise Financial Strategies’ were seldom the primary focus, but were frequently incorporated within multi-strategy interventions (Table 2)

**Table 2:** Number of studies distributed in ERIC clusters in diabetes care interventions at the PHC level in LMICs

ERIC Cluster	No. of Studies	Study Designs	How Strategies Were Implemented	Representative Study Examples
Use Evaluative and Iterative Strategies	**13**	4 quasi-experimental, 3 mixed-methods, 3 cohort/programme evaluations, 3 qualitative	Embedded audit–feedback loops, registry or EMR-based monitoring, supervisory reviews, and iterative practice refinement. Evaluation is often paired with guideline reinforcement or mobile data-entry systems.	[31, 32, 34, 38, 45, 51, 72, 89, 96, 99, 100, 108, 109].
Provide Interactive Assistance	**7**	3 quasi-experimental, 2 mixed-methods, 2 qualitative	Real-time mentoring or behavioural coaching delivered through MI, WhatsApp case support, nurse-led insulin titration protocols, or app-based follow-up. Integrated within routine PHC workflows.	[28, 63, 79, 80, 101, 110, 118]
Develop Stakeholder Interrelationships	**8**	3 RCTs, 2 mixed-methods, 2 qualitative, 1 implementation pilot	Structured coordination mechanisms linking PHC with hospitals, pharmacists, and community actors. Used shared care plans, referral pathways, and digital communication loops.	[53, 54, 67, 68, 111, 112, 114, 115]
Train and Educate Stakeholders	**10**	3 RCTs, 2 mixed-methods, 4 quasi-experimental, 1 qualitative	Skills-based provider training using checklists, structured patient education modules, refresher workshops, or SMS-based microlearning. Some included motivational interviewing or health-literacy tools.	[26, 48, 59, 64, 83, 84, 87, 92, 94, 107]
Support Clinicians	**7**	2 RCT/controlled evaluations, 3 quasi-experimental, 2 programme pilots	Digital or paper-based decision-support tools embedded in PHC workflows; task-sharing with nurses/pharmacists; teleconsultation to reinforce clinical decisions.	[31, 32, 43, 61, 72, 81, 108]
Engage Consumers	**22**	11 RCTs, 5 quasi-experimental, 2 mixed-methods, and other designs	Peer-led education, family-supported self-management, motivational interviewing, SMS/WeChat follow-up, culturally tailored dietary and lifestyle programmes.	[27, 33, 46, 56–58, 60, 62, 66, 69–71, 73, 75, 77, 78, 82, 97, 103, 104, 106, 117]
Change Infrastructure	**19**	5 quasi-experimental, 4 implementation pilots, 3 cohort evaluations, 6 mixed-methods	Introduced EMRs, chronic-care registries, structured care pathways, expanded PHC teams, decentralised medication refills, or integrated hypertension–diabetes services.	[29, 34, 37, 39–42, 45, 51, 55, 65, 86, 88, 89, 93, 96, 99, 102, 109]
Adapt and Tailor to Context	**0** (as primary strategy)	—	Contextual tailoring occurred across clusters (e.g., pictorial materials, language adaptation, culturally aligned diet counselling), but no study used this as the lead implementation strategy.	Examples embedded in other clusters [27, 66, 107].
Use Financial Strategies	**0** (as primary strategy)	—	Financial elements (e.g., bundled payments, small incentives, PHC contracting) appeared within other clusters but not as stand-alone strategies.	[32, 48, 84], (embedded financing mechanisms)

Below, we present a descriptive synthesis of how these clusters were operationalised. Studies often employed multiple strategies; to minimise repetition, we synthesise by cluster and use brief cross-referenced examples.

### Implementation strategies by clusters

#### Use evaluative and iterative strategies

Thirteen studies embedded audits, feedback, and adaptive learning cycles to strengthen diabetes care [31, 32, 34, 38, 45, 51, 72, 89, 96, 99, 100, 108, 109]. Most were quasi-experimental or mixed-methods studies and institutionalised tools such as patient registries, dashboards, routine supervision, and regular data review meetings. Representative models included the CARRS programme, which used electronic registries and nurse-coordinated audits and was associated with reductions in cardiometabolic risk factors [32], feedback loops following community screening clinics in South Africa, which improved detection and referral continuity [108], and registry-linked supervision meetings in Malaysia and Bangladesh that supported adherence to diabetes care standards [34, 96]. Digital approaches ranged from mobile decision-support systems with performance feedback mDSS [38] to serious-game–based continuing medical education [68] and integrated platforms (e.g. SMARTDiabetes) [115] that linked analytics to ongoing evaluation and quality improvement cycles [100]. In contrast, hybrid paper–digital systems and designs dependent on stable internet connectivity were more fragile and difficult to sustain [51, 89, 99].

Barriers and facilitators to implementation were closely linked to design simplicity, organisational support, and system readiness. Implementation was facilitated when evaluative strategies were low in complexity, well integrated into existing workflows (e.g. EMR prompts, call reminders), and aligned with national or programme-level policies such as WHO-PEN. In contrast, weak supply chains and limited availability of diagnostics disrupted feedback cycles and reduced continuity [100, 108]. Leadership engagement and clear ownership of registries supported accountability and routine data use, whereas staff turnover and documentation fatigue reduced follow-through [34, 96]. At the individual level, digital literacy and perceived usefulness of tools influenced fidelity [38, 72]. Programmes that formalised Plan–Do–Study–Act (PDSA) cycles and provided regular supervisory mentoring sustained learning and adaptation over time, while irregular supervision limited iterative improvement [32, 109].

Evaluative and iterative strategies were reported to support adoption, fidelity, and quality of care when they were simple, provider-led, and policy-supported while resource-intensive or connectivity-dependent systems were reported to be more difficult to sustain beyond pilot phases.

### Provide interactive assistance

Seven studies implemented real-time, bidirectional assistance for providers/patients [28, 63, 79, 80, 101, 110, 118]. These studies were conducted across China, Malawi, South Africa, Mexico, Brazil, and Thailand and used a range of interactive modalities embedded within primary healthcare delivery.

Approaches included EMR-embedded decision trees with scheduled follow-ups [79, 80]. WhatsApp-based remote mentoring for clinical officers [28], nurse-led insulin initiation with app-linked titration and specialist oversight [118], and CHW accompaniment providing structured check-ins that improved retention and HbA1c among patients with poor glycaemic control [110]. Behavioural support strategies, such as Motivational Interviewing and health-coaching delivered during routine consultations, were also used, although their impact on HbA1c varied across settings [63, 101].

Barriers and facilitators to implementation were strongly influenced by how well interactive tools were integrated into routine care. Feasibility and acceptability were higher when assistance was aligned with existing workflows and delivered through low-friction, familiar platforms such as WhatsApp [28, 80]. Policy scaffolding, including China’s Family Doctor Contract, reinforced continuity and legitimised ongoing provider–patient interaction [79]. In contrast, connectivity challenges, interruptions in medicine or supply availability, unclear role boundaries, and the absence of fidelity monitoring reduced consistency and impact [63, 101, 110, 118].

Interactive assistance supported adoption and adherence when embedded within team-based primary healthcare and reinforced through sustained mentoring or supervision. Effects on glycaemic outcomes were more variable and appeared to depend on implementation fidelity and broader system stability.

### Develop stakeholder interrelationships

Eight studies focused on strengthening coordination and partnerships across primary healthcare actors to improve continuity, accountability, and integration of diabetes care [53, 54, 67, 68, 111, 112, 114, 115]. Most were conducted in China (*n* = 5) and Mongolia (*n* = 2) and included three randomised controlled trials, two mixed-methods studies, and two qualitative studies.

Interventions commonly linked national non-communicable disease (NCD) policy to primary care delivery through structured supervision, referral coordination, and audit cycles, which supported adherence to diabetes care guidelines [53]. Physician–pharmacist partnerships improved medication management and patient counselling and were associated with better glycaemic and blood pressure control [111, 112]. Shared electronic systems and formal care contracts integrated PHC with hospital services, strengthening continuity of care and reducing unnecessary hospital utilisation [67, 114]. Educational interventions such as gamified continuing medical education (InsuOnline) improved provider knowledge and interprofessional communication [68].

Barriers and facilitators to implementation were shaped by organisational alignment and relational dynamics. Joint care pathways facilitated implementation, shared information platforms, and structured referral mechanisms that improved coordination across providers [67, 112, 112]. Policy endorsement legitimised collaboration and clarified roles, although urban–rural inequities and low literacy limited reach in some settings [111]. Within facilities, trust, task-sharing, and protected time for supervision supported teamwork, whereas hierarchical structures and staff turnover undermined collaboration [53, 111, 112]. Interactive learning approaches and peer mentorship encouraged engagement, while digital unfamiliarity and time constraints reduced consistency of participation [68, 115].

Formalised and policy-anchored stakeholder partnerships supported adoption, acceptability, and sustainability of diabetes care interventions. In contrast, weak relational infrastructure and organisational instability limited continuity and long-term integration.

### Train and educate stakeholders

Ten studies focused on strengthening workforce capability through multimodal training approaches [26, 48, 59, 64, 83, 84, 87, 92, 94, 107]. These included three randomised controlled trials, two mixed-methods studies, four quasi-experimental studies, and one qualitative study conducted across Africa, South Asia, and Latin America. Most studies reported both clinical outcomes and implementation outcomes, including effectiveness, adoption, and fidelity.

Training approaches varied in intensity and format. Skills-based workshops incorporating case discussions and role-play improved provider confidence and adherence to diabetes care protocols [26, 94]. Training that combined clinical guidelines with communication components, such as Motivational Interviewing or shared decision-making, enhanced counselling quality and patient satisfaction [84, 107]. Low-cost modular approaches, including SMS-based learning, brief refresher sessions, and implementation-intention worksheets, supported continuity and scalability of training [83, 87]. In contrast, one-off lectures delivered without follow-up or mentoring resulted in short-lived improvements [64, 92].

Barriers and facilitators to implementation were closely related to training design and organisational support. Implementation was facilitated when training was pragmatic, aligned with routine workflows, and supported by job aids, checklists, or digital modules that reinforced learning in practice [59, 87]. Policy support legitimised task-sharing and helped protect time for learning [48, 84], although shortages of diagnostics or medicines limited translation of training into clinical gains in some settings [26]. Leadership engagement promoted ownership and participation, whereas frequent staff turnover and high workload reduced attendance and continuity. Interactive and reflective training methods supported sustained behaviour change, while gaps in knowledge reinforcement and lack of supervision contributed to skill drift over time.

Training strategies improved effectiveness and adoption when they were iterative, mentored, policy-backed, and embedded within daily clinical work. Isolated or one-off training approaches had limited and short-lived impact.

### Support clinicians

Seven studies focused on supporting clinicians through strategies targeting clinical decision-making, standardisation of care, supervision, and feedback mechanisms [31, 32, 43, 61, 72, 81, 108]. These interventions aimed to strengthen provider performance and consistency of diabetes care delivery in primary healthcare settings.

Clinical Decision Support Systems (CDSS) and mobile decision-support systems (mDSS) were associated with improvements in diagnostic accuracy, treatment intensification, and adherence to clinical guidelines [32, 43, 81]). Task-sharing models, including nurse- or pharmacist-led care, ed feasibility and adoption in settings where physician capacity was limited [31, 61, 72]. Mobile screening initiatives and teleconsultation models extended clinical oversight to underserved or remote populations, supporting referral and follow-up [32, 108].

Barriers and facilitators to implementation were closely related to system simplicity and contextual fit. Implementation was facilitated when digital tools were simple, intuitive, and integrated into routine workflows, and when clinician support was reinforced through mentoring or supervision. Task redistribution supported uptake where roles were clearly defined and accepted by teams. In contrast, parallel or complex digital systems struggled in high-workload environments and were vulnerable to connectivity constraints. Limited supply stability and lack of clinician buy-in further reduced fidelity and sustained use of decision-support tools [31, 43, 81].

Clinician-support strategies were associated with effectiveness, adoption, and fidelity when simple digital tools were combined with mentoring, supervision, and appropriate task redistribution. Complex systems introduced without adequate support or system readiness showed limited sustainability

### Engage Consumers

Twenty-two studies implemented patient, family, or community-centred strategies. [27, 33, 46, 56–58, 60, 62, 66, 69–71, 73, 75, 77, 78, 82, 97, 103, 104, 106, 117]. These included 7 RCTs, 10 quasi-experimental studies, and 5 mixed-methods or qualitative studies conducted across Asia, Africa, and Latin America. Most studies reported clinical outcomes (HbA1c, fasting blood glucose, blood pressure), alongside implementation outcomes such as adoption, acceptability, feasibility, and fidelity.

A wide range of engagement approaches was used. Culturally adapted counselling and peer-led group interventions consistently reported improvements in glycaemic outcomes, self-care behaviours, and medication adherence, including the Transcultural Diabetes-Specific Nutrition Algorithm (TDNA) and Motivational Interviewing–based counselling. [66]. Peer-led education improved knowledge, self-efficacy, and Quality of Life (QoL) [73, 117]. Family-centred digital interventions, particularly those delivered through familiar platforms such as WeChat, were associated with improved HbA1c and adherence [71, 77]. mHealth registries that linked patient self-monitoring with provider review strengthened follow-up processes and continuity of care [62]. In contrast, unidirectional SMS messaging was generally feasible and acceptable but showed limited impact on glycaemic outcomes when used alone [56, 57]. Studies comparing delivery models found that hybrid approaches combining digital tools with human support (e.g. peer educators, CHWs, or nurses) outperformed stand-alone apps or digital-only interventions [75, 97]. Low-technology tools such as self-management booklets, pictorial diaries, and decision aids supported patient–provider communication and understanding, particularly in low-literacy settings, but their use was constrained by limited consultation time and variable patient literacy [69, 70, 104]. Group-based models, including medication clubs and family group visits, improved retention in care and reduced clinic congestion [46, 78].

Barriers and Facilitators: Outcomes were strongly linked to cultural fit, interactivity, and use of multiple complementary components. At the outer-setting level, community mobilisation, family involvement, and alignment with local or national policies supported delivery. In contrast, gender norms, financial hardship, and weak community–facility linkages limited reach and equity. Within facilities, integration of CHWs, peer educators, and nurses improved coordination and follow-up, whereas high workload and limited supervision reduced fidelity. At the individual level, shared peer identity, family support, and visual or low-literacy tools were associated with motivation and engagement, while digital anxiety, low literacy, and intervention fatigue reduced sustained participation. Processes such as co-design with users, group feedback sessions, message analytics, and adaptive supervision helped maintain engagement, although short project durations and weak monitoring systems constrained scale-up.

Consumer-engagement strategies were broadly acceptable and frequently associated with improvements in adherence, self-care, and glycaemic outcomes, particularly when culturally adapted and delivered through hybrid models that combined digital reminders with peer or family support and structured educational materials, integrated into routine primary healthcare

### Change infrastructure

Nineteen studies enacted system redesign to embed diabetes care in routine primary healthcare [29, 34, 37, 39–42, 45, 51, 55, 65, 86, 88, 89, 93, 96, 99, 102, 109]. Most were quasi-experimental or mixed-methods studies conducted across South and Southeast Asia, Latin America, and sub-Saharan Africa. Almost all reported clinical outcomes, while approximately half additionally measured implementation outcomes such as adoption, feasibility, fidelity, or sustainability.

Infrastructure-focused strategies are commonly aimed at improving continuity, coordination, and tracking of care. Integration of electronic medical records and unique patient identifiers improved documentation quality, follow-up, and longitudinal monitoring [41]. Decentralised models combining digital tools with nurse- or CHW-led care were frequently associated with improvements in glycaemic and blood pressure control [42]. PHC case-management frameworks were generally feasible but often constrained by shortages of medicines, diagnostics, or equipment [34]. In humanitarian settings, an integrated refugee-camp model combining health promotion, screening, and continuity of care reported improvements in glycaemic outcomes and adherence [45]. At scale, a statewide programme in Brazil demonstrated reductions in fasting glucose and systolic blood pressure compared with controls [65]. In contrast, narrow technology-only or supply-only interventions implemented without concurrent workforce strengthening or governance support showed limited sustainability [39, 99].

Barriers and facilitators to implementation reflected interactions between system design, organisational context, and governance. At the intervention level, implementation was facilitated when infrastructure changes were simple, interoperable, and aligned with existing workflows, whereas complex or reagent-dependent systems increased costs and staff burden [89, 99]. At the outer-setting level, alignment with national policy or donor priorities and reliable procurement systems supported continuity, while stock-outs, reimbursement delays, and weak governance disrupted service delivery [39, 45]. Within facilities, leadership engagement, teamwork, and shared accountability facilitated coordination and uptake, whereas staff turnover, heavy workload, and hierarchical decision-making reduced participation and fidelity [37, 86, 96, 102]. At the individual level, provider motivation and patient trust supported engagement, while burnout, low digital literacy, and scepticism regarding free medicines limited sustained use [39, 45]. Ongoing supervision, audit–feedback mechanisms, and registry-to-action loops facilitated adaptation and learning, whereas irregular monitoring or premature scale-up led to fragmented implementation [34, 42, 109].

Infrastructure change strengthened continuity, coordination, and clinical effectiveness when designs were context-fit, team-based, and anchored in supportive policy and governance environments. Digital systems and supervision were most effective when used as enabling components within broader system redesign rather than as stand-alone solutions.

### Adapt and tailor to context

No study identified contextual adaptation as the primary implementation strategy; however, elements of adaptation were commonly embedded across other strategy clusters. Studies adapted education, tools, and delivery approaches to local language, literacy, cultural norms, and resource constraints [27, 58, 66, 73, 77, 107]. Examples included culturally tailored nutrition and lifestyle counselling that reported improvement in adherence [27, 66]. low-cost behavioural approaches such as Motivational Interviewing and implementation-intention strategies that were scalable across settings [77, 107], and co-designed materials developed with communities (e.g. Mayan peer education) that supported adoption and sustainability [73].

Although not used as a stand-alone strategy, adaptation supported acceptability and contextual fit across training, consumer-engagement, and infrastructure-focused interventions.

### Utilise financial strategies

No intervention used financial strategies as the primary implementation approach; however, financial elements were frequently embedded within broader programmes to support access, adoption, and sustainability [48, 59, 85]. These included contracting arrangements, subsidies, performance-linked incentives, and reimbursement mechanisms. Examples included bundled payments under China’s Family Doctor Contract to support continuity of chronic care [48], performance-based contracts in South Africa [85], and facility-level incentives or budget earmarking to improve data completeness and follow-up [59]. Many programmes relied on short-term donor funding or pilot-based financing with limited financial autonomy, which constrained scalability and long-term sustainability.

Even modest and predictable primary healthcare financing helped stabilise implementation achieved through other strategy clusters, whereas the absence of durable financing threatened continuity and scale-up.

### Co-occurrence of ERIC clusters across programmes

**Fig. 3:**
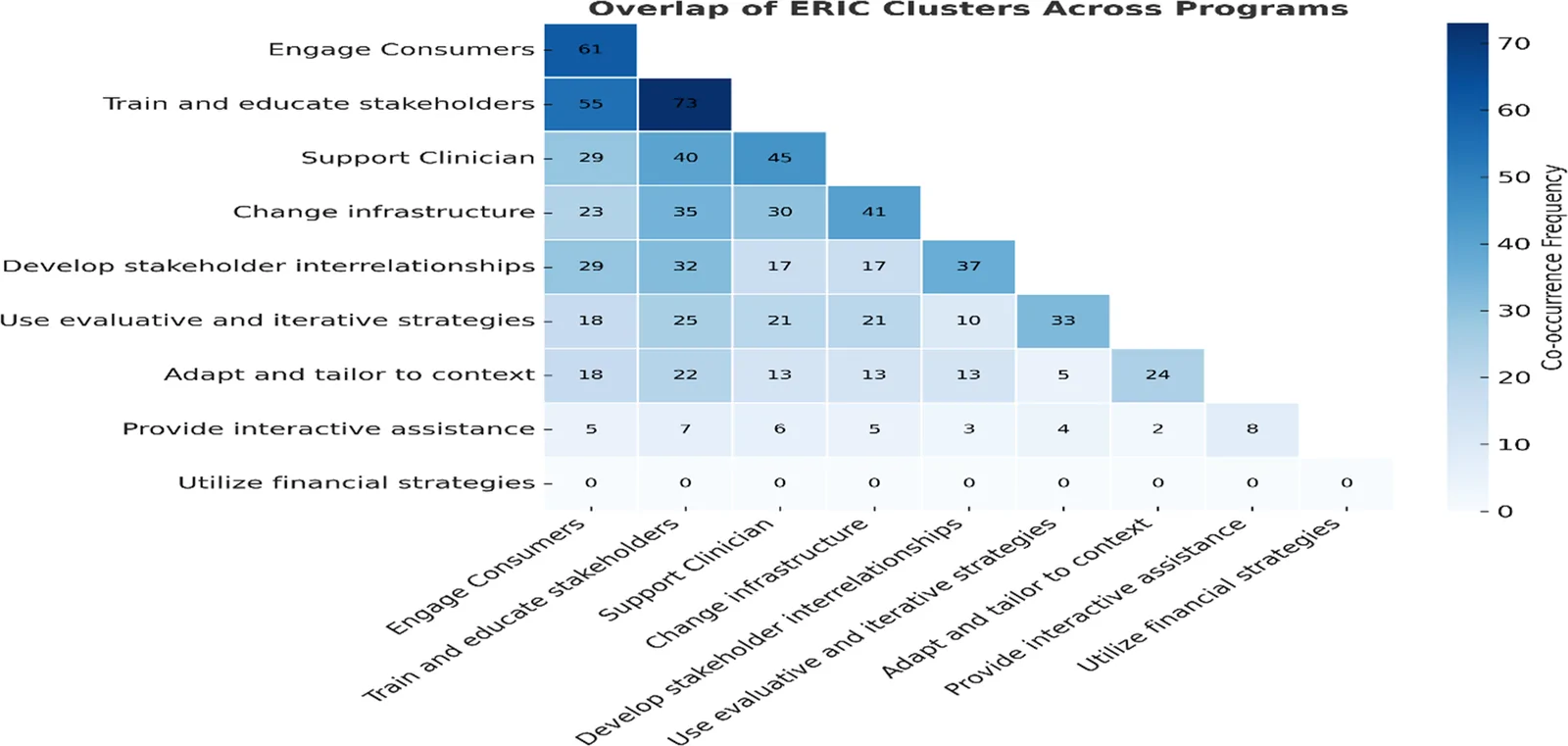
This figure illustrates the distribution of studies across ERIC clusters for diabetes care implementation in LMIC primary healthcare settings

Figure 3 illustrates the frequent co-application of implementation strategies across programmes. (Table 3) The most common pairing was Train and Educate Stakeholders × Engage Consumers, reflecting concurrent investment in provider capacity and patient activation. Train and Educate Stakeholders × Support Clinicians was also frequently observed, indicating that training was often reinforced through supervision, mentoring, or decision-support tools. Change Infrastructure commonly overlapped with Support Clinicians and Use Evaluative and Iterative Strategies, suggesting that system redesign was typically accompanied by continuous monitoring and quality improvement processes. In contrast, Adapt and Tailor to Context and Utilise Financial Strategies were less often explicitly paired with other clusters, likely reflecting limited reporting of contextual adaptation and economic planning rather than their true absence in practice.

**Table 3:** Cross-cluster overlap of implementation strategies

Strategy Pair	Co-occurrence (n)	Interpretation
Train and Educate Stakeholders × Engage Consumers	**54**	Most common combination: education efforts frequently extended to patient/family/community-facing engagement.
Train and Educate Stakeholders × Change Infrastructure	**35**	Training is often accompanied by system restructuring to operationalise new workflows.
Train and Educate Stakeholders × Support Clinicians	**34**	Training is commonly paired with decision-support or task-shifting to sustain clinical performance.
Train and Educate Stakeholders × Develop Stakeholder Interrelationships	**32**	Collaborative/team-based models relied on shared training to align cadres.
Engage Consumers × Develop Stakeholder Interrelationships	**29**	Community/peer involvement was frequently embedded within broader teamwork or referral structures.
Engage Consumers × Support Clinicians	**27**	Patient engagement strategies are often linked with provider counselling or coaching support.
Change Infrastructure × Support Clinicians	**26**	Digital/registry systems were commonly introduced alongside decision support for frontline staff.
Change Infrastructure × Engage Consumers	**23**	System redesigns frequently included patient self-management components (e.g., care plans, registries).
Adapt and Tailor to Context × Train and Educate Stakeholders	**22**	Contextual tailoring primarily occurred through culturally and linguistically adapted training content.
Train and Educate Stakeholders × Use Evaluative and Iterative Strategies	**21**	Audit–feedback and monitoring cycles were most commonly implemented where training was central.
Adapt and Tailor to Context × Engage Consumers	**18**	Tailoring was especially used in community-based and peer-led self-management programmes.
Change Infrastructure × Develop Stakeholder Interrelationships	**17**	Organisational reforms often required inter-team coordination or governance structures.
Develop Stakeholder Interrelationships × Support Clinicians	**17**	Multidisciplinary case-review, referral linkage, and shared-care strengthened clinician support.
Provide Interactive Assistance × others	**Low (1–4 each)**	Used selectively, typically layered onto digital or community-supported models.
Utilise Financial Strategies × others	**Very low (<3 each)**	Financial components were embedded rather than primary strategies; rarely coordinated systematically.

### Synthesis across studies

Across LMIC primary healthcare settings, implementation of diabetes care appeared more successful when multiple ERIC strategy clusters were combined to address clinical, organisational, and contextual barriers. Provider-focused strategies, including training, decision support, and team-based care, strengthened clinical capability and care processes. Consumer-engagement strategies supported adherence and self-management, particularly when culturally adapted and linked to routine PHC follow-up. Infrastructure change and iterative evaluation reported improvement in feasibility and sustainability when supported by reliable supply chains, supervision, and governance mechanisms.

Synthesis of barriers and facilitators using CFIR domains highlighted the importance of alignment between adaptable intervention design, leadership and teamwork within facilities, and supportive policy and community environments. Overall, implementation approaches that were context-responsive, iterative, and multi-level, simultaneously strengthening providers, engaging patients, and reinforcing health system infrastructure, were more likely to support sustained improvements in diabetes care (Table 4)

**Table 4:** CFIR – barriers and facilitators to implementation of diabetes interventions in LMIC primary care

CFIR Domain	Facilitators	Barriers	Representative Studies
Intervention Characteristics	Structured, evidence-based guidelines and protocols facilitated provider buy-in	Outdated or poorly aligned guidelines limited adherence	[35, 53] [31, 45, 58] [59, 63] [38, 65]
	Digital tools (mHealth apps, SMS, CDSS) supported adherence and self-management	Technological complexity, poor internet access, and phone-sharing undermined effectiveness	
	Culturally adapted tools (visual aids, simplified messaging, peer-led support) were associated with patient engagement	High cost of devices/medicines and lack of trained specialists (e.g., dieticians, foot care)	
	Point-of-care diagnostics and simplified monitoring logbooks reported improvement in continuity	Variability in intervention delivery and limited infrastructure reduced fidelity	
Outer Setting	National and district-level policy support and WHO-endorsed guidelines enabled adoption	Competing health priorities and weak guideline alignment limited continuity	[35, 53, 59] [34, 45, 65], [45, 58, 63] [93, 114]
	Community mobilisation and partnerships with NGOs or hospitals strengthened delivery	Medication shortages and poor diagnostic infrastructure undermined service delivery	
	High mobile phone access and digital outreach supported intervention reach	Socioeconomic barriers (affordability, transport, insurance gaps) restricted access	
	Trust in family doctors and community health systems reported improvement in adherence	Low patient trust in PHC and reliance on hospitals weakened uptake	
Inner Setting	Strong leadership support at facility and district levels enabled programme integration	Weak leadership and a lack of accountability hindered continuity	[35, 53, 119] [31, 59], [41, 45, 58, 65] [34, 63, 89]
	Task redistribution and multidisciplinary teamwork reported improvement in service delivery	High staff turnover, workload pressures, and insufficient incentives disrupted delivery	
	EMR integration and registry systems strengthened data tracking and follow-up	Duplication of records, administrative inefficiencies, and resource shortages reduced effectiveness	
	Ongoing supervision, embedded training systems, and mentoring reported improvement in fidelity	Limited infrastructure, physical space, and logistical capacity constrained implementation	
Characteristics of Individuals	Training reported improvement in provider confidence, skills, and motivation	Knowledge gaps and resistance among staff to new roles limited uptake	[31, 59, 63] [35, 53, 58] [45, 56, 77], [65, 73, 78]
	Motivational interviewing and culturally sensitive education increased empathy and patient engagement	Low patient literacy, poor health awareness, and reluctance to adopt behavioural changes reduced impact	
	Patients valued personalised counselling, goal setting, and visual aids for self-management	Socioeconomic constraints, psychological distress, and gender norms weakened participation	
	Peer educators empowered low-literacy populations and reported improvement in engagement	Low trust in PHC services and poor continuity undermined sustained involvement	
Process	Stakeholder engagement and partnerships reported improvement in responsiveness and local adoption	Weak coordination across levels of government and poor accountability limited fidelity	[35, 45, 53] [58, 59, 63] [31, 45, 65] [45, 58, 63]
	Supervision, refresher training, and structured feedback loops associated with implementation quality	Inconsistent supervision and the absence of feedback systems reduced fidelity	
	Participatory approaches (e.g., action research, family involvement, embedded local actors) strengthened community engagement	High attrition, selective delivery, and short project duration undermined sustainability	
	Digital tools and WhatsApp-based case discussions streamlined clinical support and peer exchange	Incomplete tool implementation and inconsistent data collection hindered long-term monitoring	

## Discussion

This scoping review mapped diabetes care interventions’ strategies in low- and middle-income countries (LMICs) to the Expert Recommendations for Implementing Change (ERIC) taxonomy, identifying how strategies were used to strengthen primary healthcare for chronic disease management. Although country income status may change over time, the majority of included studies were conducted in the past decade, limiting the impact of such transitions on the interpretation of findings. Interpretation of findings should consider variation across urban and rural settings, but only a subset of studies explicitly reported. However, both rural and urban contexts were represented, with a slightly higher proportion conducted in rural settings. This inconsistent reporting of settings across studies limits detailed comparison. Across 85 studies, interventions addressed seven ERIC clusters, most frequently Train and Educate Stakeholders, Change Infrastructure, and Provide Interactive Assistance [31, 37, 110]. In contrast, no studies explicitly prioritised Adapt and Tailor to Context or Utilise Financial Strategies as stand-alone strategies, although contextual and financial elements were often embedded within broader programmes [45, 66]. While the ERIC taxonomy provides a structured approach to categorising implementation strategies, its generic nature may limit the ability to capture the specific clinical components of diabetes care being implemented. Diabetes management in primary healthcare involves multiple domains, including glycaemic control, cardiovascular risk reduction, complication screening, and behavioural support. Many of the strategies identified in this review, such as task-sharing, simplified clinical algorithms, team-based care, and use of non-laboratory-based risk assessment tools, align closely with World Health Organisation recommendations for diabetes care in resource-constrained settings. Future implementation research would benefit from explicitly linking implementation strategies to specific components of diabetes care to enhance relevance for health system decision-making.

Most interventions sought to strengthen delivery system design and self-management support, typically through provider training, workflow reorganisation, and patient education or digital tools [40, 115]. These reflect the reliance of LMIC health systems on frontline providers and patient engagement in long-term care. Infrastructure changes such as electronic medical records, point-of-care diagnostics, and outreach programmes were widely reported [41, 42]. Interactive assistance and clinician support, often delivered via mentoring, decision-support systems, and telemonitoring, underscored the growing role of digital innovations, though uptake varied [81, 101].

A consistent gap was the limited use of explicit contextual adaptation. While some interventions employed culturally tailored materials or peer-led approaches [73, 97]. These were rarely evaluated as systematic adaptation processes. Financial strategies such as incentives, reimbursement, or pooled funding were largely absent, despite frequent reports of cost and sustainability challenges [105, 108]. This misalignment between recognised implementation frameworks and financing realities highlights a critical area for future intervention design.

Mapping to the Consolidated Framework for Implementation Research (CFIR) revealed cross-cutting determinants. At the intervention level, guideline-based tools, digital platforms, and culturally adapted materials facilitated uptake [35, 96], while intervention complexity, literacy demands, and device costs posed barriers [38, 62].

At the outer setting level, supportive national policies, NGO partnerships, and community mobilisation enabled scale-up [56, 105]. Yet frequent medication stock-outs, diagnostic gaps, and transport or insurance challenges undermined continuity [34, 45].

Within the inner setting, leadership engagement, electronic records, and multidisciplinary teamwork supported implementation [85, 95]. Conversely, staff turnover, workload, and administrative inefficiencies remained persistent barriers [36, 88].

Individual characteristics also influenced outcomes: provider confidence after training and patient willingness to adopt new roles acted as enablers [42, 56], while low literacy and weak motivation limited intervention effectiveness. Finally, process-level facilitators included participatory engagement, supervision, and feedback loops [60, 117], but fidelity often faltered due to inconsistent follow-up or shortened interventions [50, 104].

Few reviews have systematically examined the implementation of diabetes care in LMIC primary healthcare. One review of 198 studies on hypertension and diabetes found that most strategies focused on service organisation (76.5%), commonly delivered by non-specialists (43.4%) or multidisciplinary teams (25.5%) [120]. This aligns with our finding that delivery system redesign and task redistribution dominate LMIC strategies. However, while Correia’s review classified interventions by type (screening, QI, integrated services), our ERIC-based synthesis provides granularity by identifying the actual implementation strategies applied.

Other reviews focusing on specific domains reinforce our findings. Mokaya et al. synthesised mHealth interventions for type 2 diabetes in LMICs [121], reporting feasibility and engagement benefits but highlighting persistent barriers of digital literacy, access, and affordability, echoing our CFIR-based findings [38, 62]. Fitzpatrick’s review of diabetes self-management education (DSME) programmes concluded that while acceptable and effective in improving knowledge, such interventions often lacked robust reporting on adoption, fidelity, and cost [122]. This resonates with our observation that contextual adaptation and financial sustainability remain underused.

Regionally focused reviews also identified similar challenges. In a scoping review of West African diabetes care, the authors highlighted systemic barriers such as weak infrastructure, inadequate financing, and limited governance [123]. These determinants mirror the outer and inner setting challenges identified in our broader LMIC sample.

Together, existing reviews highlight the predominance of training and service delivery redesign in LMIC diabetes care, while consistently noting gaps in contextual adaptation, financing, and sustainability. This review extends the literature by moving beyond intervention typologies to show how implementation strategies are combined in practice, which strategies tend to co-occur, and under what contextual conditions they are more likely to be adopted, sustained, or disrupted. By linking strategies to reported implementation and clinical outcome signals, this synthesis provides actionable insights for policymakers and implementers seeking to design context-responsive, multi-component approaches for strengthening diabetes care in primary healthcare settings.

The following policy and practice implications were derived directly from the cross-cluster synthesis of implementation strategies, outcome patterns, and reported barriers and facilitators. Specifically, priorities were identified based on strategies that were most frequently associated with positive implementation or clinical outcome signals across multiple studies, as well as recurrent constraints that limited adoption, fidelity, sustainability, or scale-up. These implications, therefore, reflect areas where the existing evidence suggests the greatest potential leverage for strengthening diabetes care implementation in LMIC primary healthcare settings.:

1. **Financial sustainability**: Few studies explicitly used financial strategies, yet costs, stock-outs, and workforce incentives repeatedly emerged as barriers. Embedding reimbursement for expanded roles (e.g., pharmacists, CHWs), securing reliable medication supply chains, and allocating budget lines for chronic care at the PHC level are essential for lasting impact.
2. **Systematic contextual adaptation**: While culturally tailored materials and peer support reported improvement in engagement [66, 73]. These adaptations were often ad hoc. Policymakers can institutionalise adaptation by promoting co-design with communities, translating guidelines into local languages, and embedding flexible delivery mechanisms.
3. **Integration into PHC systems**: Effective strategies such as audit and feedback loops [95], EMR and registry tools [41, 80], and outreach models [88] worked best when linked to district leadership and national programmes. Institutionalising these mechanisms beyond project pilots will enhance continuity.
4. **Community and family engagement**: Interventions using family support via WeChat [71] or peer-led education [33, 60] reported improvement in adherence and behaviour change. Formal integration of CHWs, peer groups, and family counselling into PHC diabetes programmes can strengthen sustainability.

### Strengths and limitations

Strengths: This scoping review was conducted using a comprehensive and systematic approach. We searched eight bibliographic databases and relevant grey literature, including studies published in languages other than English, and applied transparent eligibility and screening procedures. Implementation strategies were systematically mapped using the ERIC taxonomy, while barriers and facilitators were synthesised using CFIR to support structured, context-sensitive interpretation. Reporting followed the PRISMA-ScR checklist, enhancing transparency and reproducibility. (Annexure 4)

Limitations: This scoping review has several limitations that should be considered when interpreting the findings. These relate both to the review methodology and to the nature and reporting of the included studies.

First, we did not undertake a formal quality appraisal or risk-of-bias assessment. This decision is consistent with scoping review methodology, which aims to map the extent and characteristics of the evidence rather than to assess effectiveness or study quality. Consequently, the findings should be interpreted as descriptive and exploratory, and not as evaluations of comparative effectiveness.

Second, the review relied primarily on published literature, which may have introduced publication bias, as studies reporting successful or positive implementation experiences are more likely to be published. Although grey literature was searched, unpublished programme reports and routine implementation experiences may be under-represented.

Third, while a transparent and systematic approach was used, the classification of dominant implementation strategies required interpretive judgement, particularly for multi-component interventions. Although this was mitigated through co-occurrence analysis and narrative synthesis, some subjectivity is inherent in mapping complex interventions to taxonomy-based frameworks.

Fourth, a small number of non-English studies (5 in number) were included for full-text review. Translation was primarily conducted using machine translation tools; one included study for data extraction was verified by a native speaker for accuracy of translation. Although this approach enhanced accessibility, there remains a possibility of minor inaccuracies in interpretation.

Limitations of the included studies also affected the synthesis. There was substantial heterogeneity in study design, intervention complexity, outcome selection, and reporting, which limited direct comparability across studies. Implementation outcomes such as adoption, fidelity, acceptability, and sustainability were inconsistently defined and often reported descriptively rather than using standardised measures.

Few studies examined long-term sustainability, scale-up, or maintenance, and economic considerations, such as cost or cost-effectiveness, were rarely reported. This constrained insights into the durability and affordability of implementation strategies in resource-constrained primary healthcare settings. Finally, many studies prioritised clinical outcomes, with limited reporting on implementation processes, contextual adaptations, or mechanisms of change, restricting deeper understanding of how and why strategies worked in specific contexts

## Conclusion

Diabetes care interventions in LMIC primary healthcare most frequently relied on provider training, infrastructure changes, and interactive assistance, while explicit contextual adaptation and financial strategies were rarely the primary focus. Implementation was commonly reported to be supported by leadership engagement, use of digital tools, and community involvement, but constrained by supply shortages, staff turnover, and weak feedback mechanisms.

By mapping implementation strategies and synthesising reported barriers and facilitators, this review provides a detailed overview of how diabetes care has been implemented in LMIC primary healthcare settings and the contextual factors that influence implementation processes. The findings highlight priorities for future implementation efforts, including embedding financial sustainability, strengthening integration within PHC systems, adopting more systematic approaches to contextual adaptation, and positioning community engagement as a core component of diabetes care. Addressing these areas may support a shift from short-term projects toward more resilient, equitable, and sustainable models of chronic disease management in LMICs

## Electronic supplementary material

Below is the link to the electronic supplementary material.

Supplementary Material 1

Supplementary Material 2

Supplementary Material 3

Supplementary Material 4

## Data Availability

All data generated or analysed during this study are included in this published article and its supplementary information files.
