# Supplementary material for: Implementation strategies for evidence-based management of type 2 diabetes at primary healthcare facilities in low- and middle-income countries and related barriers and facilitators: a scoping review
Source: Implement Sci Commun. 2026 Jun 16;7:167. doi: 10.1186/s43058-026-00998-9 (PMC13508276; doi:10.1186/s43058-026-00998-9)
Supplement: Supplementary file 1 [file 43058_2026_998_MOESM1_ESM.docx]

1.1. **Stage 1**

The search strategy aims to identify keywords and index terms specific to each information source. A preliminary limited search of PubMed and Google Scholar has informed this. The identified articles are assessed for the text words and index terms used in the titles and abstracts.

**Relevant MeSH Terms and Keywords**

Diabetes Mellitus

- MeSH Term: "Diabetes Mellitus"[MeSH]
- Keywords: diabetes, diabetes mellitus, diabetic

Primary Healthcare

- MeSH Term: "Primary Health Care"[MeSH]
- Keywords: primary healthcare, primary care, primary health care, general practice, family practice

Low- and Middle-Income Countries

- MeSH Term: "Developing Countries"[MeSH]
- Keywords: low-income countries, middle-income countries, LMIC, developing countries, resource-limited settings, <https://zenodo.org/records/8019937>

Implementation Strategies

- MeSH Term: "Implementation Science"[MeSH], "Health Plan Implementation"[MeSH]
- Keywords: implementation strategies, implementation science, program implementation, dissemination, knowledge translation, adoption, diffusion of innovation, implementation.
- Eric Strategies: [ERIC strategy chain.docx](https://1drv.ms/w/s!AmCLSRMV00hJgfZZA-54o90tqqEwbQ?e=jg6jze)

Barriers and Facilitators

- Key Words: barrier*, facilitat*, enable*, influenc*

Explanation of the Use of Asterisks:

- Diabet*: Captures diabetes, diabetic, diabetics, etc.
- Primary Health*: Covers both "primary health care" and "primary healthcare."
- Practice*: Includes both "practice" and "practices."
- Countr*: Retrieves "country" and "countries."
- LMIC*: Captures LMIC (Low and Middle Income Countries) and its plural form.
- Setting*: Covers both "setting" and "settings."
- Strateg*: Includes "strategy," "strategies," etc.
- Implement*: includes “implementation” and “Implemented” and “implementation” and “implementing” etc.
- Barrier*: retrieves both barrier and barriers
- Facilitat*: includes facilitators, facilitation, facilitating etc
- Influenc*: includes influencers, influencing

Implement* ADJ2 strateg*: Captures "implementation strategy", "implementation strategies" and “implementing strategy” ensuring the words "implementation" and "strategy/strategies" are within two words of each other.

[Scoping Review Search String.docx](https://1drv.ms/w/s!AmCLSRMV00hJgfZGTxf-AH_2rrRg7g?e=buBYmG)

1.2. **Stage 2**

In this stage, all identified keywords and index terms will be administered across all included databases.

1.3. **Stage 3**

Lastly, this search strategy will target the additional sources in the references list of finalised reports and article

**Database: Embase <1996 to 2024 Week 35>, Ovid MEDLINE(R) <1996 to August Week 5 2024> Search Strategy:**

**1**  ("Diabetes Mellitus MeSH Terms" or diabet* or diabetes mellitus or diabetic*).mp. [mp=ti, ab, hw, tn, ot, dm, mf, dv, kf, fx, dq, bt, nm, ox, px, rx, ui, sy, ux, mx] (2058588)

**2**  ("Primary Health Care MeSH Terms" or primary healthcare or primary health* care or primary care or general practice* or family practice*).mp. [mp=ti, ab, hw, tn, ot, dm, mf, dv, kf, fx, dq, bt, nm, ox, px, rx, ui, sy, ux, mx] (530750)

**3**  ("Developing CountriesMeSH Terms" or low-income countr* or middle-income countr* or LMIC* or developing countr* or resource-limited setting* or afghan* or africa* or albania* or algeria* or angola* or antigua* or barbuda* or argentin* or armenia* or aruba* or azerbaijan* or bahrain* or bangladesh* or bengal* or bangal* or barbados* or barbadian* or bajan or bajans or belarus* or belorus* or byelarus* or byelorus* or belize* or benin* or dahomey or bhutan* or bolivia* or bosnia* or herzegovin* or botswan* or batswan* or bechuanaland* or brazil* or brasil* or bulgaria* or burkina* or burkinese* or upper volta* or burundi* or urundi* or cabo verde* or cape verde* or cambodia* or kampuchea* or khmer* or cameroon* or cameroun* or ubangi shari* or chad* or chile* or china* or chinese or colombia* or comoro* or comore* or comorian* or mayotte* or congo* or zaire* or costa rica* or "cote d'ivoir*" or "cote d' ivoir*" or cote divoir* or cote d ivoir* or ivory coast* or ivorian* or croatia* or cuba or cuban or cubans or "cuba's" or cyprus* or cypriot* or czech* or djibouti* or french somaliland* or dominica* or ecuador* or egypt* or united arab republic* or el salvador* or salvadoran* or guinea* or equatoguinea* or eritrea* or estonia* or eswatini* or swaziland* or swazi* or swati* or ethiopia* or fiji* or gabon* or gabonese* or gabonaise* or gambia* or ((georgia or georgian or georgians) not (atlanta or california or florida)) or ghana* or gibraltar* or greece* or greek* or grecian* or grenada* or grenadian* or guam* or guatemala* or guyana* or guiana* or guyanese* or haiti* or hispaniola* or hondura* or hungary* or hungarian* or india* or indonesia* or iran* or iraq* or isle of man* or jamaica* or jordan* or kazakh* or kenya* or karabati* or korea* or kosovo* or kosova* or kyrgyz* or kirgiz* or kirghiz* or laos or lao or laotian* or latvia* or lebanon* or lebanese* or lesotho* or lesothan* or lesothonian* or basutoland* or mosotho* or basotho* or liberia* or libya* or jamahiriya* or lithuania* or macedonia* or madagasca* or malagasy* or malawi* or nyasaland* or malaysia* or malay* federation or maldives* or maldivian* or indian ocean or mali or malian* or "mali's" or malta or maltese* or "malta's" or micronesia* or marshallese* or kiribati* or marshall island* or nauru or nauran or nauruans or "naurian's" or mariana or marianas or palau or paluan* or tuvalu* or mauritania* or mauritan* or mauritius* or mexico* or mexican* or moldova* or moldovia* or mongol* or montenegr* or morocco* or moroccan* or ifni or mozambique* or mozambican* or myanmar* or burma* or burmese or namibia* or nepal* or new caledonia* or netherlands antill* or nicaragua* or niger* or oman or omani or omanis or "oman's" or pakistan* or palestin* or gaza* or west bank* or panama* or paraguay* or peru or peruvian* or "peru's" or philippine* or philipine* or phillipine* or phillippine* or filipino* or filipina* or poland* or polish or pole or poles or portugal* or portuguese or puerto ric* or romania* or russia* or ussr* or soviet* or rwanda* or rwandese or ruanda* or ruandese or samoa* or navigator island* or pacific island* or polynesia* or sao tome* or santomean* or saudi arabia* or saudi or saudis or senegal* or serbia* or seychell* or sierra leone* or slovak* or sloven* or melanesia* or solomon island* or norfolk island* or somali* or sri lanka* or ceylon* or saint kitts or st kitts or kittian* or nevisian* or saint lucia* or st lucia* or saint vincent* or st vincent* or vincentian* or grenadine* or sudan* or surinam* or syria* or tajik* or tadjik* or tadzhik* or tanzania* or tanganyika* or thai* or timor leste* or east timor* or timorese* or togo or togoles* or "togo's" or tonga* or trinidad* or tobago* or tunisia* or turkiy* or turkey* or turk or turks or turkish or turkmen* or uganda* or ukrain* or uruguay* or uzbek* or vanuatu* or new hebrides* or venezuela* or vietnam* or viet nam* or yemen* or yugoslav* or zambia* or zimbabwe* or rhodesia* or arab* countr* or middle east* or global south or sahara* or subsahara* or magreb* or maghrib* or west indies* or caribbean* or central america* or latin america* or south america* or asia central or central asia* or asia northern or north asia* or northern asia* or asia southeastern or southeastern asia* or south eastern asia* or southeast asia* or south east asia* or asia western or west asia* or western asia* or europe eastern or east europe* or eastern europe* or developing countr* or developing nation* or developing population* or developing world or less developed countr* or less developed nation* or less developed world or lesser developed countr* or lesser developed nation* or lesser developed world or under developed countr* or under developed nation* or under developed world or underdeveloped countr* or underdeveloped nation* or underdeveloped world or middle income countr* or middle income nation* or middle income population* or low income countr* or low income nation* or low income population* or lower income countr* or lower income nation* or lower income population* or underserved countr* or underserved nation* or underserved population* or under served population* or under served nation* or under served population* or deprived countr* or deprived population* or high burden countr* or high burden nation* or countdown countr* or countdown nation* or poor countr* or poor nation* or poor population* or poor world or poorer countr* or poorer nation* or poorer population* or poorer world or developing econom* or less developed econom* or underdeveloped econom* or under developed econom* or middle income econom* or low income econom* or lower income econom* or low gdp or low gnp or low gross domestic or low gross national or lower gdp or lower gnp or lower gross domestic or lower gross national or lmic or lmics or third world or lami countr* or transitional countr* or emerging econom* or emerging nation*).mp. [mp=ti, ab, hw, tn, ot, dm, mf, dv, kf, fx, dq, bt, nm, ox, px, rx, ui, sy, ux, mx] (6022324)

**4**  (((implement* or implementation science or implement*) adj2 strateg*) or program implementation or dissemination or knowledge translation or adoption or diffusion of innovation).mp. [mp=ti, ab, hw, tn, ot, dm, mf, dv, kf, fx, dq, bt, nm, ox, px, rx, ui, sy, ux, mx] (439853)

**5**  ("Access new funding" or "Alter incentive*" or "Alter patient fees" or "Assess readiness" or "Audit ADJ2 feedback" or "Build coalition" or "Capture ADJ2 knowledge" or "Centralize technical assistance" or "Change accreditation" or "Change liability laws" or "Change physical structure" or "Change record system*" or "Change service sites" or "Conduct small tests" or "Conduct educational meetings" or "Educational outreach" or "Local consensus discussions" or "Needs assessment" or "Ongoing training" or "Learning collaborative" or "New clinical teams" or "Change credentialing" or "Implementation blueprint" or "Academic partnerships" or "Implementation glossary" or "Quality monitoring" or "Organize monitoring systems" or "Develop disincentives" or "Educational materials" or "Resource sharing" or "Distribute materials" or "Relay ADJ2 data" or "Facilitation" or "Fund clinical innovation" or "Prepare champions" or "Identify early adopters" or "Increase demand" or "Inform opinion leaders" or "Enhance ADJ2 adherence" or "Involve executive boards" or "Involve patients" or "Make billing easier" or "Dynamic training" or "Mandate change" or "Simulate change" or "Patient ADJ2 feedback" or "Formal commitments" or "Clinician team meetings" or "Fee for service" or "Prepare patients" or "Promote adaptability" or "Network weaving" or "Clinical supervision" or "Local technical assistance" or "Ongoing consultation" or "Reexamine implementation" or "Train leadership" or "Remind clinicians" or "Revise roles" or "Shadow experts" or "Stage scale-up" or "Dissemination organization" or "Tailor strategies" or "Advisory boards" or "Implementation advisor" or "Capitated payments" or "Data experts" or "Data warehousing" or "Mass media" or "Other payment schemes" or "Train-the-trainer" or "Visit other sites" or "Work with educational institutions").mp. [mp=ti, ab, hw, tn, ot, dm, mf, dv, kf, fx, dq, bt, nm, ox, px, rx, ui, sy, ux, mx] (211404)

**6**  (barrier* or facilitat* or enabler* or influencer*).mp. [mp=ti, ab, hw, tn, ot, dm, mf, dv, kf, fx, dq, bt, nm, ox, px, rx, ui, sy, ux, mx] (2222989)

**7**  4 or 5 or 6 (2716450)

**8**  1 and 2 and 3 and 7 (1496)
