# Supplementary material for: Implementation strategies for evidence-based management of type 2 diabetes at primary healthcare facilities in low- and middle-income countries and related barriers and facilitators: a scoping review
Source: Implement Sci Commun. 2026 Jun 16;7:167. doi: 10.1186/s43058-026-00998-9 (PMC13508276; doi:10.1186/s43058-026-00998-9)
Supplement: Supplementary file 2 [file 43058_2026_998_MOESM2_ESM.docx]

**General information**

**Study ID / Source**

Database, Peer reviewed journal / grey literature

**Title**

Title of paper / abstract / report that data are extracted from

**Year of Publication**

**Type of article (original, policy document, conference proceeding, report etc)**

**Lead author contact details**

**The country in which the study was conducted**

please choose "other" if not in the list or more than one country.

1. China
2. India
3. Malaysia
4. Mexico
5. Pakistan
6. South Africa
7. Thailand
8. Other

Clear above selection

**Type of Primary Healthcare Unit**

**Single Center / Multicenter**

**Characteristics of included studies**

**Methods**

**Aim of study**

**Study Design**

**Start date**

**End date**

**Study funding sources**

**Participants**

**Inclusion criteria**

**Exclusion criteria**

**Method of recruitment of participants**

1. Phone
2. Mail
3. Clinic patients
4. Voluntary
5. Other

Clear above selection

**Total number of participants**

**Data collection methods or tools**

**Baseline Population Characteristics**

|  | **Intervention 1** | **Intervention 2** | **Comparison** | **Overall** |
| --- | --- | --- | --- | --- |
| **Age** |  |  |  |  |
| **Gender** |  |  |  |  |
| **Duration of diabetes** |  |  |  |  |
| **any comorbidity** |  |  |  |  |
| **HbA1c at baseline** |  |  |  |  |
| **Any other characteristic** |  |  |  |  |

**Intervention and Comparisons**

|  | **Intervention 1** | **Intervention 2** | **Comparison** |  |
| --- | --- | --- | --- | --- |
| **name of intervention** |  |  |  |  |
| **Who delivered the intervention** |  |  |  |  |
| **material used** |  |  |  |  |
| **mode of delivery** |  |  |  |  |
| **tools of assessment** |  |  |  |  |
| **duration of the intervention** |  |  |  |  |
| **training of provider** |  |  |  |  |
| **content of intervention** |  |  |  |  |
| **any other detail** |  |  |  |  |

**ERIC strategy category**

For details on ERIC Strategies please follow the link below

https://1drv.ms/w/c/4948d31513498b60/Ea1Tc5ynTBxJjtxyroOAbsMBkuF0nY9rWnAtETS3V8ej1Q?e=WWkYcE

1. Financial Strategies
2. Infrastructure Strategies
3. Supporting Clinicians
4. Educational Strategies
5. Patient-Facing Strategies
6. Policy and Regulation Strategies
7. Quality Management
8. Community Engagement
9. Adaptation and Flexibility

Clear above selection

**Detail of the selected ERIC strategy (Adapted/replicated/novel)**

**Type of outcomes, clinical / implementation / both**

**Outcome table (Time Point 1)**

|  | **HbA1c** | **outcome 2** | **outcome 3** | **outcome 4** |
| --- | --- | --- | --- | --- |
| **Intervention 1 Mean (SD)/% 0r OR** |  |  |  |  |
| **Intervention 2Mean (SD)/% 0r OR** |  |  |  |  |
| **Comparison Mean (SD)/% 0r OR** |  |  |  |  |

**Outcome table (Time Point 2)**

|  | **HbA1c** | **Outcome 2** | **Outcome 3** | **Outcome 4** |
| --- | --- | --- | --- | --- |
| **Intervention 1 Mean (SD)/% 0r OR** |  |  |  |  |
| **Intervention 2 Mean (SD)/% 0r OR** |  |  |  |  |
| **Comparison Mean (SD)/% 0r OR** |  |  |  |  |

**Qualitative outcomes**

**Implementation outcome**

**Facilitators**

**Barriers**

**Main findings/suggestions of the study**

**Limitations and strengths of the study**
