# Supplementary material for: Implementation strategies for evidence-based management of type 2 diabetes at primary healthcare facilities in low- and middle-income countries and related barriers and facilitators: a scoping review
Source: Implement Sci Commun. 2026 Jun 16;7:167. doi: 10.1186/s43058-026-00998-9 (PMC13508276; doi:10.1186/s43058-026-00998-9)
Supplement: Supplementary file 3 [file 43058_2026_998_MOESM4_ESM.docx]

# PRISMA-ScR Checklist

**Title of Manuscript: Implementation Strategies for evidence-based diabetes management at primary healthcare facilities in low- and middle-income countries and related barriers and facilitators: A scoping review**

| **Section** | **Item** | **PRISMA-ScR item** | **Location in manuscript / How addressed** |
| --- | --- | --- | --- |
| **Title** | 1 | Identify the report as a scoping review | The title explicitly identifies the study as a scoping review. |
| **Abstract** | 2 | Structured summary | The abstract provides a structured summary including background, objectives, eligibility criteria, information sources, data charting methods, synthesis approach, key results, and conclusions. |
| **Introduction** | 3 | Rationale | The rationale for conducting a scoping review is described, highlighting the growing burden of diabetes in LMICs, the importance of primary healthcare, and gaps in the implementation of evidence-based diabetes care. |
|  | 4 | Objectives | The objectives are clearly stated: to identify implementation strategies for diabetes care in primary healthcare settings in LMICs, categorise strategies using the ERIC taxonomy, and map reported barriers and facilitators using the CFIR framework. |
| **Methods** | 5 | Protocol and registration | A review protocol was developed a priori and registered with the Open Science Framework (OSF). |
|  | 6 | Eligibility criteria | Eligibility criteria included LMIC setting (World Bank classification), primary healthcare context, diabetes-related interventions, and eligible study designs. |
|  | 7 | Information sources | Eight electronic databases were searched, covering publications from January 2000 to November 2024. |
|  | 8 | Search strategy | The full electronic search strategy is provided in an appendix. The search strategy was peer-reviewed using the PRESS checklist. |
|  | 9 | Selection of sources of evidence | Titles, abstracts, and full texts were independently screened by two reviewers using Covidence, with disagreements resolved through discussion. |
|  | 10 | Data charting process | Data were extracted using a structured and piloted charting form developed and applied in Covidence. |
|  | 11 | Data items | Extracted data included study characteristics, country, primary healthcare setting, intervention details, implementation strategies (ERIC), barriers and facilitators (CFIR), and reported clinical and implementation outcomes. |
|  | 12 | Critical appraisal of individual sources | Critical appraisal was not conducted, consistent with the objectives of a scoping review, and this is explicitly stated in the manuscript. |
|  | 13 | Synthesis of results | Results were synthesised narratively and organised by ERIC implementation strategy clusters, with barriers and facilitators mapped to CFIR domains. |
| **Results** | 14 | Selection of sources of evidence | The number of records identified, screened, included, and excluded (with reasons) is reported using a PRISMA flow diagram generated from Covidence. |
|  | 15 | Characteristics of sources of evidence | Characteristics of included studies (e.g. country, primary healthcare setting, study design, intervention type) are summarised in tables. |
|  | 16 | Results of individual sources of evidence | Each included study was mapped to relevant ERIC implementation strategies and CFIR constructs. |
|  | 17 | Synthesis of results | Common implementation strategies, co-occurring strategy patterns, and key contextual barriers and facilitators are summarised across studies. |
| **Discussion** | 18 | Summary of evidence | The discussion summarises key patterns in implementation strategies, contextual determinants, and reported outcomes relevant to diabetes care in LMIC primary healthcare settings. |
|  | 19 | Limitations | Study limitations are acknowledged, including heterogeneity of included studies and the absence of formal quality appraisal. |
|  | 20 | Conclusions | Conclusions highlight implications for the design, implementation, and scale-up of sustainable diabetes care interventions in LMIC primary healthcare systems. |
| **Funding** | 21 | Funding | This study forms part of a doctoral research programme funded by the Centre for IMPACT, University of York. The funder had no role in the design of the study; data collection, analysis, or interpretation; or the writing of the manuscript |
